# Analytical Form of the Fluorescence Correlation Spectroscopy Autocorrelation Function in Chemically Reactive Systems

**DOI:** 10.1021/acs.jctc.3c01176

**Published:** 2024-03-22

**Authors:** Andrzej Poniewierski, Robert Hołyst

**Affiliations:** Institute of Physical Chemistry, Polish Academy of Sciences, Kasprzaka 44/52, Warsaw 01-224, Poland

## Abstract

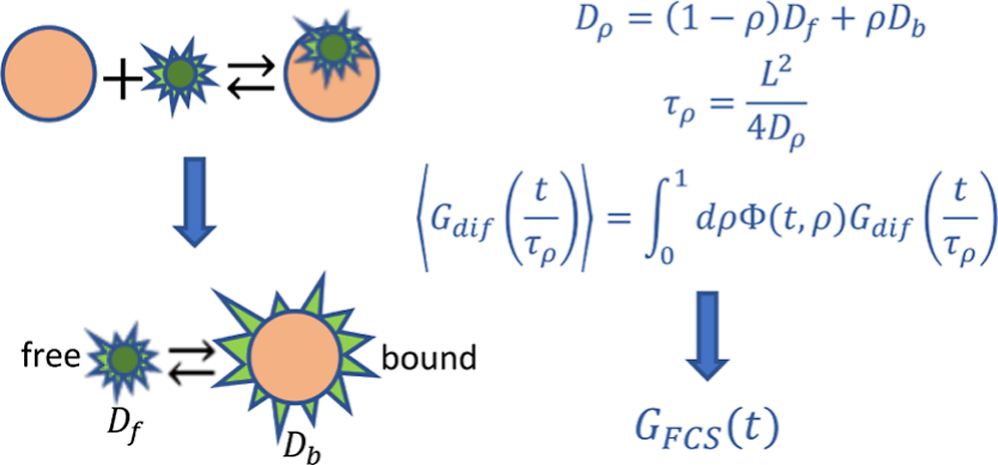

Fluorescence correlation spectroscopy (FCS) applied to chemically reactive systems provides information about chemical reaction equilibrium constants and diffusion coefficients of reactants. These physical quantities are determined from the FCS-measured autocorrelation function, *G*(*t*), as a function of time, *t*. In most of the studied cases, the analytical form of *G*(*t*) is well-known for reactions that are much faster than the diffusion time of reactants across the focal volume probed by FCS or when they are much slower than the diffusion time. Here, we develop an analytical form of *G*(*t*) for reactions occurring at an intermediate time scale comparable to the diffusion time. *G*(*t*) depends on the reaction rates in such reactions. We focus on reversibly binding a fluorescently labeled small molecule to a macromolecule in a diluted solution in thermodynamic equilibrium. Our approach allows the analysis of FCS data over a wide range of diffusion coefficients, reaction rate constants, and brightness levels of fluorescent labels. Our *G*(*t*) is valid even when the fluorescent label changes its brightness upon binding. The easy-to-implement analytical form of the autocorrelation function greatly helps experimentalists study chemical reactions, determining the equilibrium constants of reactions and the reaction rates.

## 1 Introduction

Fluorescence correlation spectroscopy (FCS) is a helpful method for monitoring the relaxation of concentration fluctuations in ideal solutions at thermodynamic equilibrium. Magde, Elson, and Webb first used FCS to study the reversible binding of ethidium bromide (EtBr) to DNA.^1^ EtBr is a dye that forms a strongly fluorescent complex with DNA. Local component concentrations fluctuate around equilibrium concentrations due to reaction and diffusion processes, resulting in a coupling between the two processes. Consequently, the decay of the concentration fluctuations depends on the reaction rate constants, equilibrium concentrations, and diffusion coefficients. All these parameters appear in the fluorescence fluctuation autocorrelation function, *G*(*t*), whose time dependence is determined experimentally by FCS. The conceptual and theoretical foundations of FCS, as well as experimental methods and problems, were presented by Elson and Magde in separate articles.^2,3^ For more discussion of this technique and its variants, see, e.g., refs (4–8). A comprehensive review of the FCS applications in analytical chemistry, biophysics, and cell biology was given by Krichevsky and Bonnet.^9^ The great advantage of FCS is that it does not require any external perturbations of the tested system. For example, researchers have used this method to study rotational diffusion,^10^ dynamics of cyclodextrin-pyronine complexation,^11^ microsecond dynamics of proteins,^12^ intermolecular interactions between dyes and nucleotides,^13^ and protein–DNA interactions.^14^ Recent examples of FCS applications to analyze cell biochemical processes can be found in refs (15–18).

In this theoretical work, we focus on the applications of FCS to chemically reactive equilibrium systems where nonfluorescent macromolecules and dye molecules form fluorescent complexes. Some time ago, we studied noncovalent interactions between surfactant micelles and fluorescent dyes using the FCS method.^19^ Unlike the DNA–EtBr complex, whose fluorescence is much greater than that of the free dye, the fluorescence of the micelle-bound dye did not change upon binding. Due to the coupling of reaction and diffusion processes, deriving a general expression for *G*(*t*) in closed form is a real challenge. The standard approach to the problem is diagonalizing the reaction–diffusion (RD) equations expressed in Fourier space. Using this method, we derived an approximate formula for *G*(*t*),^20^ which we applied successfully to the system experimentally studied in ref (19). However, our approximation contained the exponential integral, *E*_1_(*z*), which is not an elementary function and therefore does not satisfy the definition of a closed-form expression.

Over the years, much attention has been devoted to investigating the above-mentioned systems using FCS. For example, Michelman-Ribeiro et al.^21^ studied the association and dissociation rates of DNA binding in live cells. They adopted a two-component model in which the labeled molecules diffuse freely or are bound to immobile binding sites. Thus, the diffusion coefficient of the bound component is zero. Four simplified regimes were defined on the plane spanned by reaction rate constants: pure diffusion, effective diffusion, hybrid model, and reaction-dominant regime. In the case of pure or effective diffusion, *G*(*t*) is approximated by the autocorrelation function for single-component diffusion with the appropriate diffusion coefficient. The hybrid model is used when the labeled molecules’ binding rate to immobile binding sites is much greater than the detachment rate. Then, *G*(*t*) is approximated by an integral over the wave vector, which is not an elementary function. Finally, in the reaction-dominant regime, the diffusion time of labeled molecules is much shorter than that of binding to binding sites. Then, *G*(*t*) is approximated by combining autocorrelation functions for free and bound molecules. Wirth, Ludes, and Swinton used a similar model in the context of fluorophores interacting with heterogeneous chemical interfaces.^22^ It is also worth noting that similar problems were studied using fluorescence recovery after photobleaching (FRAP).^23−26^ An example is the diffusion of messenger molecules in the presence of traps, for which both FCS and FRAP can be used.^27−31^

Our current goal is to derive a closed-form expression for *G*(*t*) that could be used in FCS studies of the above-mentioned chemical system in a wide range of reaction rate constants and diffusion coefficients. Moreover, it should apply regardless of the relationship between the fluorescence of the free dye and the complex. To simplify the problem, one usually assumes that the diffusion coefficients of the macromolecule and complex are equal. Then, the RD equations for the original system can be transformed into another set of RD equations. One of the new equations corresponds to the free diffusion of the macromolecule, while the other two are formally identical to the RD equations for unimolecular isomerization.^2,9^ The latter has a simple solution when the diffusion coefficients of the two species are equal. In the case under study, however, the macromolecule and dye diffusion coefficients are usually very different. Then, the diagonalization of the RD equations leads to a complex dependence of the eigenvalues and eigenvectors on the wave vector, which causes inherent difficulties in integrating over the wave vector. Therefore, we propose a different approach to this problem. We present the solution of the RD equations as the diffusion of infinitely many virtual components whose diffusion coefficients assume unique values between the value for the macromolecule and the dye. The diffusion of each component resembles free diffusion but with an amplitude dependent on time and reaction rate constants. At first glance, our approach is more complicated than the standard method. However, it leads to a Gaussian form of the solution with respect to the wave vector, as in the case of free diffusion. Consequently, we get an expression for *G*(*t*) that contains only algebraic and exponential dependence on time.

The plan of our paper is as follows. In Section 2, we define the model and then transform the RD equations as described above. From the solution of the transformed equations, we derive the general strict form of *G*(*t*) as a functional of the autocorrelation function for single-component diffusion. We then define approximations to obtain analytical expressions for this functional. Section 3 derives the autocorrelation function for fast, slow, and intermediate-rate reactions using an appropriate approximation. In particular, we show that the autocorrelation functions for effective diffusion and two-component diffusion of noninteracting species are reproduced. The former applies when the chemical reaction is much faster than the complex and dye diffusion time scales. We then determine the applicability range of the approximation by comparing the approximate *G*(*t*) with exact numerical calculations. Our approximation can be used for intermediate-rate reactions even if the reaction and diffusion time scales are comparable. We also consider the fast diffusion regime and the situation where the reaction rates of dye binding and detachment are very different. In Section 4, we provide a summary of our method to facilitate its implementation in FCS experiments. Finally, we dedicate Section 5 to discussion and conclusions.

## 2 Model

We consider a dilute solution, in thermodynamic equilibrium, of macromolecules, *A*, fluorescent dyes, *B*, and fluorescent complexes, *C*. The latter is the product of a binary reaction
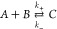
1where *k*_+_ and *k*_–_ are the reaction rate constants for the forward and backward reactions, respectively. FCS experiments study the autocorrelation function of fluorescence fluctuations, , where δ*n*(*t*) is the fluctuation in the number of collected photons, *n*(*t*). The ensemble average *n̅* = ⟨*n*(*t*)⟩ does not depend on time in equilibrium systems. In turn, δ*n*(*t*) is related to the kinetics of concentration fluctuations that occur in the observation volume due to the chemical reaction and diffusion. The evolution of these small fluctuations results from linearized RD equations
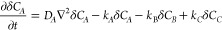
2a
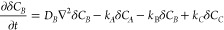
2b
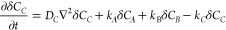
2cwhere , *C*_*i*_(***r***, *t*) is the local concentration of component *i* = *A*, *B*, and *C*, and  is its equilibrium concentration. For brevity, we denote , , and *k*_*C*_ = *k*_–_. All diffusion coefficients are usually different, but if the dye molecule is small, it can be assumed that *D*_*A*_ = *D*_*C*_ = *D* ≪ *D*_*B*_.

The RD model deals with noninteracting point particles in solution. This model is consistent with the FCS theory that assumes the ideality of the solution tested by this technique. In an ideal solution, the correlation length of equilibrium concentration fluctuations is much smaller than the average distance between solute particles. Therefore, interactions between them are neglected. From the point of view of the FCS technique, the assumption of a point particle is justified when the fluorescent particle is small compared to the linear size of the focal volume. The dye distribution within the particle should also be considered in the case of a large particle. The issue of submicrometer particles comparable to the size of the focal volume is discussed, for example, by Deptuła et al.^32^

The relationship between *G*(*t*) and solutions of eqs 2 is derived in ref (9). Therefore, we focus only on the main points. *G*(*t*) is a combination of concentration fluctuations’ auto- and cross-correlation functions, ⟨δ*C*_*i*_(***r***, 0)δ*C*_*j*_(***r***′, *t*)⟩, convoluted with the excitation light distribution, *I*(***r***). The latter is usually in the Gaussian form
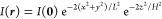
3where *L* is the beam radius and *H* is the axial dimension measured in the direction of light propagation. The aspect ratio ω = *H*/*L* is usually larger than 1. The issue of approximating the light intensity distribution in the focal spot with a Gaussian profile is also discussed in ref (32). The FCS autocorrelation function has the following form
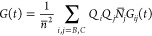
4where *Q*_*B*_ and *Q*_*C*_ are the quantum yields of the fluorescent components,  is the mean number of the type *i* molecules in the observation volume *V*, and . Finally, *G*_*ij*_(*t*) are the components of *G*(*t*), which we define below.

To derive *G*_*ij*_(*t*) from the solution of eqs 2, we use the Green’s function method. For example, Green’s function for a freely diffusing particle, , is the probability density that, at time *t*, the particle will be at ***r*** if it initially was at ***r***′. Using this function, Agmon derived the conditional residence probability that plays the role of the autocorrelation function in the single-molecule FCS.^33^ For the system studied here, the general solution of eqs 2 is expressed in terms of Green’s functions as follows
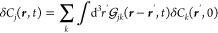
5where δ*C*_*k*_(***r***′, 0) is the concentration fluctuation at *t* = 0 and *j*, *k* = *A*, *B*, and *C*. For each value of *k*, Green’s functions, , must satisfy eqs 2 with the initial conditions , where δ_*jk*_ is the Kronecker delta and δ(***r*** – ***r***′) is the Dirac δ function. Then,  because the initial positions of different molecules are not correlated and the concentration fluctuations are subject to Poisson statistics. The relation between *G*_*ij*_(*t*), Green’s functions, and the excitation light distribution is
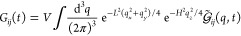
6where  is the Fourier transform of . *V* = π^3/2^*L*^2^*H* is called the effective sampling volume.^9^ Then, *G*_*ij*_(0) = δ_*ij*_ due to the initial conditions .

### 2.1 Exact Results

Equations 2 are usually expressed in Fourier space and solved by standard matrix diagonalization. However, the dependence of eigenvalues and eigenvectors on *q* is relatively complex. Therefore, integrating over the wave vector in eq 6 cannot be done analytically unless the eigenvalues and eigenvectors are approximated.^28−31^ To avoid this problem, we express , , and  using new functions , , and  as follows
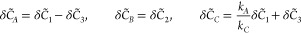
7

Transformation (7) leads to a new set of RD equations
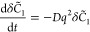
8a
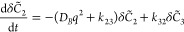
8b
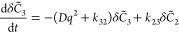
8cwhere *k*_23_ = *k*_*B*_ and *k*_32_ = *k*_*A*_ + *k*_*C*_ are the reaction rate constants for a fictitious reaction
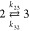
9

Equation 8a corresponds to the free diffusion of species 1. Equations 8b and 8c are the RD equations for species 2 and 3, respectively, characteristic of single-molecule isomerization with different isomer diffusion coefficients. In our case, species 2 and 3 correspond to the dye molecule’s free and bound states, respectively. In the bound state, the dye diffuses together with the macromolecule. Then, we solve eqs 8 for the Fourier transforms of Green’s functions, , with the initial conditions , where *i*′, *j*′ = 1, 2, and 3. Hence
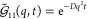
10a
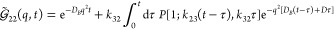
10b
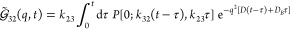
10c
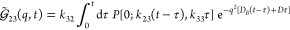
10d
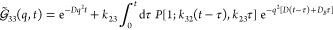
10ewhere
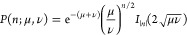
11is the Skellam distribution of the variable *n*(34) and *I*_|*n*|_(ζ) is the modified Bessel function;^35^ the remaining four Green’s functions have the value zero. We show in the Supporting Information that the pairs , , and ,  are two independent solutions of eqs 8b and 8c. It is worth noting that the Skellam distribution was used recently by Vesga et al. in an FCS study of reversible membrane–protein association.^36^ Similarly to eq 6, we define
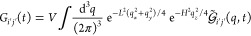
12where *i*′, *j*′ = 1, 2, and 3. The relations between *G*_*ij*_(*t*) and *G*_*i*′*j*′_(*t*) result from transformation (7). In the case of fluorescent components, these are
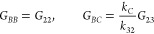
13a
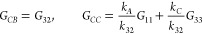
13b

It is convenient to express *G*(*t*) in a form analogous to eq 4 using *G*_*i*′*j*′_(*t*). Therefore, for species 1, 2, and 3, we define the quantum yields
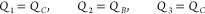
14and equilibrium concentrations
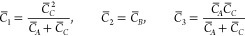
15

Since transformation (7) mixes *A* with *C*, we also treat species 1 as fluorescent. The definition of  is consistent with the reaction (9) equilibrium constant, . In addition,  and
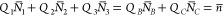
16where  for *i*′ = 1, 2, and 3 by analogy with  for *i* = *B* and *C*. However, we cannot assign different types of molecules to species 1 and 3. Each of them contains free macromolecules and macromolecule–dye complexes in different proportions, but . Combining eqs 13a–15 leads to the expression for *G*(*t*) in terms of species 1, 2, and 3
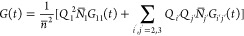
17

Due to the Gaussian dependence of  on *q*, we can express *G*_*i*′*j*′_(*t*) for *i*′, *j*′ = 2 and 3 as functionals of the autocorrelation function for single-component diffusion, i.e.
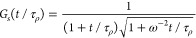
18where τ_ρ_ = *L*^2^/4*D*_ρ_ is a characteristic diffusion time across the observation volume. The diffusion coefficient *D*_ρ_ = (1 – ρ)*D*_*B*_ + ρ*D* takes values between *D* and *D*_*B*_ for 0 ≤ ρ ≤ 1. Finally, we derive the following exact expressions for *G*_*i*′*j*′_(*t*)
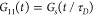
19a
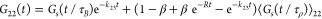
19b
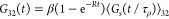
19c
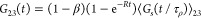
19d
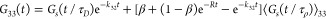
19ewhere τ_*D*_ = *L*^2^/4*D* and τ_*B*_ = *L*^2^/4*D*_*B*_ are the diffusion times, *R* = *k*_23_ + *k*_32_ is the chemical relaxation rate, and β = *k*_23_/*R*. The average values of *G*_*s*_(*t*/τ_ρ_) for *i*′, *j*′ = 2 and 3 are
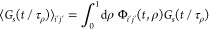
20where Φ_*i*′*j*′_(*t*, ρ) are probability density functions of ρ, which follow from eqs 10b–10e. They depend on time and the reaction rate constants, *k*_23_ and *k*_32_, whereas *G*_*s*_(*t*/τ_ρ_) depends on the macromolecule and dye diffusion coefficients. Since Φ_23_(*t*, ρ) = Φ_32_(*t*, ρ), , hence . The average value prefactors in eqs 19 are positive and monotonic functions of time that result from normalization
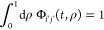
21

Explicit expressions for Φ_*i*′*j*′_(*t*, ρ) using the Skellam distribution for *n* = 0 and *n* = 1 are given in the Supporting Information. To summarize this section, eqs 17–19 are the basis for calculating *G*(*t*). They are exact, but calculating the average values of *G*_*s*_(*t*/τ_ρ_) requires approximations, which we present below.

### 2.2 Approximations

#### 2.2.1 Expansion of *G*_*s*_(*t*/τ_ρ_) around Average ρ

**Figure 1:**
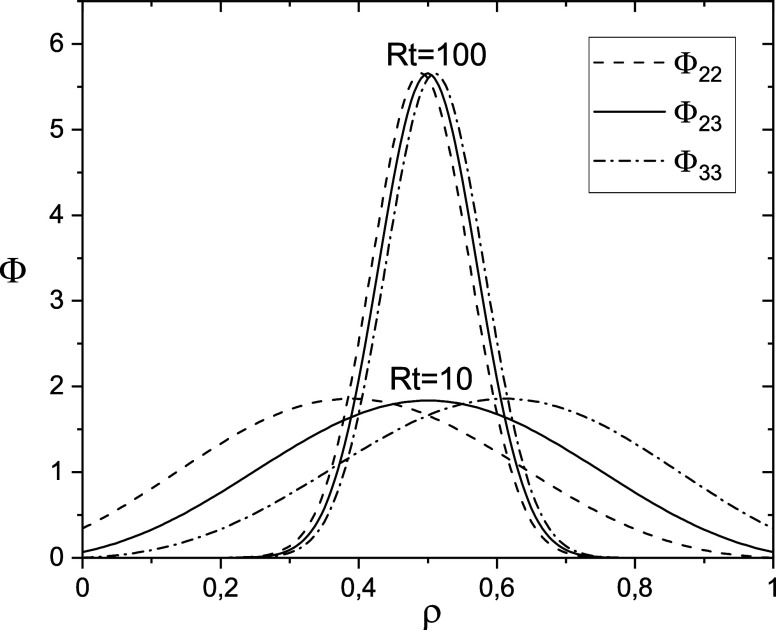
Dependence of Φ_22_, Φ_23_ = Φ_32_, and Φ_33_ on ρ for *Rt* = 10 (broad distributions) and *Rt* = 100 (narrow distributions); in both cases, β = 0.5. All probability density functions tend to δ(ρ – β) when *Rt* → ∞.

Examples of probability density functions are shown in Figure 1. When *Rt* ≫ 1, each of them has one maximum near ρ = β and tends to the Dirac δ(ρ – β) function in the limit *Rt* → ∞. This behavior results from the asymptotic expansion of *I*_*n*_(ζ),^35^ hence
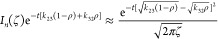
22for *Rt* → ∞, where , , *k*_23_ = *R*β, and *k*_32_ = *R*(1 – β). Therefore, the range of ρ values that contribute significantly to  shrinks as *Rt* increases. This fact suggests expanding *G*_*s*_(*t*/τ_ρ_) around ρ = β. However, our studies show that expanding it around the average ρ is better. Therefore, we use the approximation
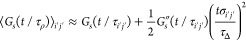
23

We have omitted the higher-order terms in ρ – ⟨ρ⟩_*i*′*j*′_ for practical reasons. The meaning of symbols: *G*_*s*_″(χ) is the second derivative of *G*_*s*_(χ)
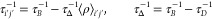
24and  is the standard deviation of Φ_*i*′*j*′_(*t*, ρ). Note that , where  is the average diffusion coefficient. Since ⟨ρ⟩_*i*′*j*′_ is a function of time, the same applies to τ_*i*′*j*′_. The key issue is deriving explicit expressions for ⟨ρ⟩_*i*′*j*′_ and . We present them in the summary section. In particular, we show that ⟨ρ⟩_*i*′*j*′_ – β and  tend to zero as (*Rt*)^−1^ when *Rt* → ∞.

#### 2.2.2 Probability Density Functions for *Rt* → 0

The form of Φ_*i*′*j*′_(*t*, ρ) for *Rt* → 0 results from the expansion of *I*_0_(ζ) and *I*_1_(ζ) for ζ → 0. We show in the Supporting Information that
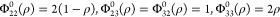
25where the superscript denotes the limit *Rt* → 0. In Section 3.3, we study the case of a slow reaction when *R* → 0 at fixed *t* and β. In particular, using definition (20) yields
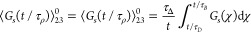
26where *G*_*s*_(χ) is defined by eq 18. We will use eq 26 in Section 3.3.

## 3 Autocorrelation Function

In this section, we apply the formalism presented in Section 2 to calculate *G*(*t*). We consider three cases: fast reaction, slow reaction, and intermediate-rate reaction. By this classification, we mean the relationship between reaction and diffusion rates. We also consider the fast diffusion limit, which is another way to study slow reactions, and the regimes given by the conditions *k*_23_ ≪ *k*_32_ and *k*_32_ ≪ *k*_23_.

### 3.1 Fast Reaction

A reaction is called fast if the diffusion of each fluorescent component is much slower than the forward and backward reactions. As *Rt* increases, all probability density functions become more concentrated around the average value of ρ. Later in this section, we will show that for sufficiently fast reactions, we can reduce expansion (23) to the first term, i.e.
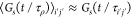
27

Therefore, by fast reaction, we mean a reaction to which approximation (27) applies. Then, eqs 19 take the following simple form
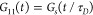
28a
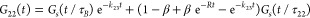
28b
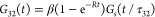
28c
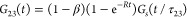
28d
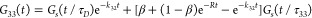
28e

The diffusion times, τ_22_, τ_33_, and τ_32_ = τ_23_, depend on *t* (see Section 4).

#### 3.1.1 Effective Diffusion

When *Rt* → ∞,  tends to the effective diffusion coefficient, *D*_β_ = (1 – β)*D*_*B*_ + β*D*. *G*(*t*) for effective diffusion depends not on the reaction rate constants but on the equilibrium constant. We obtain *G*(*t*) for this case by taking the limit *R* → ∞ at fixed β in eqs 28. Hence, *G*_22_(*t*) = *G*_23_(*t*) = (1 – β)*G*_*s*_(*t*/τ_β_) and *G*_33_(*t*) = *G*_32_(*t*) = β*G*_*s*_(*t*/τ_β_), where τ_β_ = *L*^2^/4*D*_β_. Using eqs 14–17 yields the autocorrelation function for effective diffusion
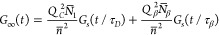
29where , , and . *Q*_β_ = *Q*_*B*_(1 – β) + *Q*_*C*_β is the effective quantum yield. Note also that . Therefore, *G*_∞_(*t*) has the same form as the autocorrelation function for independent two-component diffusion with the diffusion coefficients *D* and *D*_β_, equilibrium concentrations  and , and quantum yields *Q*_*C*_ and *Q*_β_. Equation 29 generalizes the autocorrelation function for effective diffusion in the case *Q*_*B*_ = *Q*_*C*_.

### 3.2 Intermediate-Rate Reaction

This section will consider reaction rates greater than or comparable to the diffusion rate. We will show that approximation (23) is then sufficient. The condition for the applicability of approximation (23) follows from the asymptotic behavior of the probability density functions for *Rt* → ∞ (see Section 2.2.1). When , all Φ_*i*′*j*′_(*t*, ρ) tend to δ(ρ – β). Approximation (23) is consistent with the effective diffusion limit if Φ_*i*′*j*′_(*t*, ρ) is concentrated around ρ = β for times comparable to τ_β_, i.e., if . Since τ_β_^–1^=(1 – β)τ_*B*_^–1^+βτ_*D*_^–1^, we get the inequality
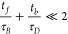
30where *t*_*f*_ = *k*_23_^–1^ and *t*_*b*_ = *k*_32_^–1^ are the relaxation times of the free and bound states of the dye. Condition (30) is satisfied if both relaxation times are small compared to the corresponding diffusion times. Then,  can be approximated by *G*_*s*_(*t*/τ_β_). However, we can refine condition (30) because approximation (23) is based on the expansion around the average ρ and not around ρ = β. For this purpose, we define the relative deviations of the approximate average values of *G*_*s*_(*t*/τ_ρ_) from the exact ones
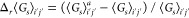
31where the superscript *a* refers to approximation (23). These relative deviations depend on the diffusion times τ_*D*_ and τ_*B*_ and the reaction rate constants *k*_23_ and *k*_32_. We set τ_*B*_ as a unit of time to reduce the number of independent parameters. Then, we define the accuracy of approximation (23), , as the maximal value of |Δ_*r*_⟨*G*_*s*_⟩_*i*′*j*′_| for all times and all values of *i*′ and *j*′. The approximation is valid if , where ϵ is the required accuracy. In this article, we assume ϵ = 0.01, which seems small enough in the context of FCS experiments. Next, we determine the range of parameters compatible with the condition . To facilitate this task, we express γ as a function of β and the parameter *v* = (*t*_*f*_/τ_*B*_) + (*t*_*b*_/τ_*D*_), i.e.
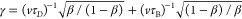
32

As γ increases, each probability density function becomes more concentrated around the average ρ, which means that  decreases and the approximation accuracy improves. Therefore, the worst accuracy corresponds to the minimum of γ(β) at *v*, τ_*D*_, and τ_*B*_ fixed. The minimum occurs at β_min_ = τ_*D*_/(τ_*D*_ + τ_*B*_) and is . Since  and γ_min_ is inversely proportional to *v*, we can find the limiting value of *v* below which . This value, denoted *v*_*L*_, depends only on the ratio τ_*B*_/τ_*D*_. As a result,  when *v* < *v*_*L*_ so that we can replace condition (30) with the inequality
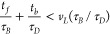
33

If condition (33) is satisfied, the accuracy of approximation (23) is better than ϵ.

**Figure 2:**
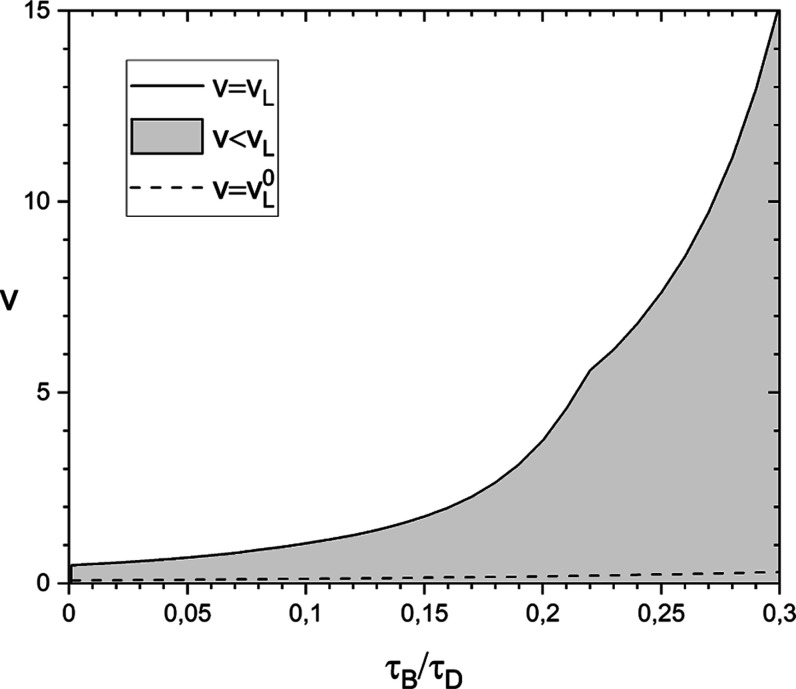
Applicability condition for the two approximations of the *G*_*s*_(*t*/τ_ρ_) average value described in the text. Approximation (23) applies in the area below the solid line. Its accuracy is better than 1% if *v* = *t*_*f*_/τ_*B*_ + *t*_*b*_/τ_*D*_ < *v*_*L*_. Approximation (27) applies in the area below the dashed line. Its accuracy is better than 1% if *v* < *v*_*L*_^0^. The lowest value of τ_*B*_/τ_*D*_ in this diagram is 0.001.

**Figure 3:**
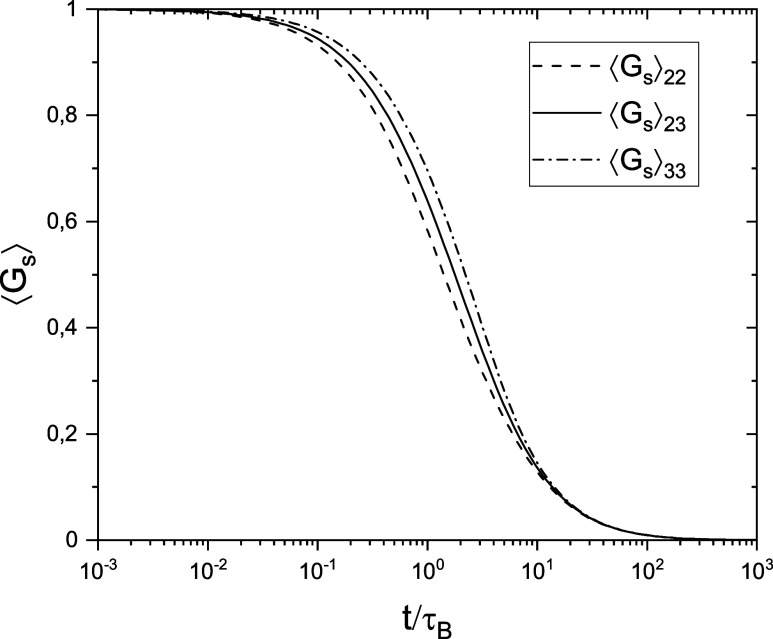
Average values of *G*_*s*_(*t*/τ_ρ_) vs time for the parameters τ_*B*_/τ_*D*_ = 1/6, β = 0.5, and *v* = 1 < *v*_*L*_(1/6). This set of parameters corresponds to *R*τ_*B*_ = 7/3.

**Figure 4:**
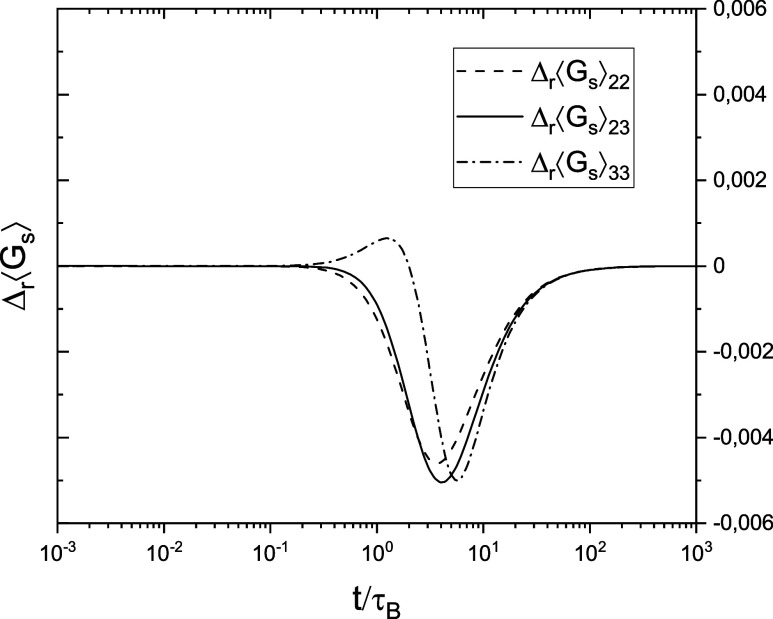
Time dependence of the relative deviations of approximate average values from exact average values calculated from approximation (23). Parameters τ_*B*_/τ_*D*_, β, and *v* are the same as in Figure 3.

We numerically determined *v*_*L*_ as a function of τ_*B*_/τ_*D*_ in the interval 0.001 ≤ τ_*B*_/τ_*D*_ ≤ 0.30. It is represented by a solid line in Figure 2. For instance, τ_*B*_/τ_*D*_ = 1/6 corresponds to *v*_*L*_ ≈ 2; hence, approximation (23) is valid if *t*_*f*_ ≲ τ_*B*_ and *t*_*b*_ ≲ τ_*D*_. In contrast, τ_*B*_/τ_*D*_ = 0.001 corresponds to *v*_*L*_ ≈ 0.48. This comparison shows that the parameter’s range that matches the condition  shrinks significantly when the macromolecule is much less mobile than the dye molecule. We return to this point in Section 3.5. In Figures 3 and 4, we show sample graphs of  and  vs time for selected parameters meeting condition (33), i.e., β = 0.5, τ_*B*_/τ_*D*_ = 1/6, and *v* = 1 < *v*_*L*_(1/6). Figure 4 clearly shows that both short-time and long-time limits of  are reproduced very well by approximation (23). The most significant deviations occur in the 1 < *t*/τ_*B*_ < 10 range but are also well below the assumed accuracy of 0.01.

We also applied a similar analysis to the case of the fast reaction described in Section 3.1 using approximation (27). The limiting value of *v*, denoted *v*_*L*_^0^, is represented by a dashed line in Figure 2. It is evident that approximation (27) is limited to fast reactions compared to the diffusion rates of both components. For example, *v*_*L*_^0^(1/6) is 1 order of magnitude smaller than *v*_*L*_(1/6).

### 3.3 Slow Reaction

By slow reaction, we mean that the diffusion of each component across the focal volume is much faster than the reaction rates, i.e., τ_*B*_ ≪ 1/*k*_23_ and τ_*D*_ ≪ 1/*k*_32_. According to the terminology used in ref (21) in the context of immobile binding sites (see also the Supporting Information), the first condition determines the reaction-dominant regime. Then, the second condition cannot be applied because τ_*D*_ is infinite.

When γ*t* is small, *I*_0_(ζ) and *I*_1_(ζ) in eqs 10 can be expanded around ζ = 0, i.e., *I*_0_(ζ) ≈ 1 and *I*_1_(ζ) ≈ ζ/2. This approximation leads to the following expressions
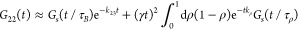
34a
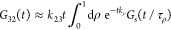
34b
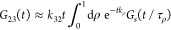
34c
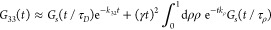
34dwhere *k*_ρ_ = (1 – ρ)*k*_23_ + ρ*k*_32_. We can easily find the lower and upper bounds of these functions because *G*(*t*/τ_ρ_) is an increasing and convex function of ρ, hence
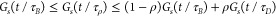
35

Therefore, the lower and upper bounds consist of exp(−*tk*_23_) and exp(−*tk*_32_) with time-dependent amplitudes. If *k*_23_*t* ≪ 1 and *k*_32_*t* ≪ 1, then exp(−*tk*_ρ_) ≈ 1, and the integrals can be calculated analytically. Hence
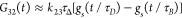
36a
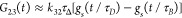
36bwhere
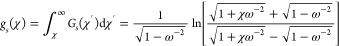
37

The terms proportional to (γ*t*)^2^ in *G*_22_(*t*) and *G*_33_(*t*) are omitted. Finally, we approximate *G*(*t*) for slow reactions and *t* ≪ γ^–1^ as follows
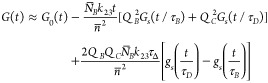
38where
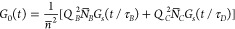
39is the autocorrelation function for the diffusion of two noninteracting species. The last two terms are corrections proportional to the reaction rate constant *k*_23_.

### 3.4 Fast Diffusion

From the formal point of view, *G*_*s*_(*t*/τ_ρ_) can also be considered the probability density of the variable ρ. Therefore, we define a normalized function
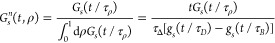
40

We will limit ourselves to immobile macromolecules, assuming *D* = 0. Hence
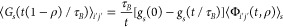
41where ⟨ ⟩_*s*_ denotes the average with *G*_*s*_^*n*^(*t*,ρ). By fast diffusion, we mean the asymptotic limit *D*_*B*_ → ∞ (τ_*B*_ → 0). Then, *G*_*s*_^*n*^(*t*,ρ) → δ(1 – ρ) and the average value of *G*_*s*_(*t*/τ_ρ_) with Φ_*i*′*j*′_(*t*, ρ) can be approximated by
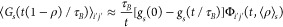
42

We use approximation (22) to calculate Φ_*i*′*j*′_(*t*, ⟨ρ⟩_*s*_) for long times compared to γ^–1^, which gives
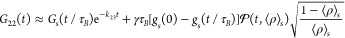
43a
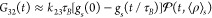
43b
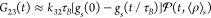
43c
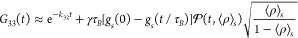
43dwhere
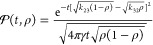
44When τ_*B*_/*t* → 0, ⟨1 – ρ⟩_*s*_ decays as , where we have ignored the amplitude dependent on the aspect ratio ω. Hence,  decays as . In the case of *G*_22_(*t*) and *G*_33_(*t*),  is multiplied by ) and ), respectively. To approximate *G*_*i*′*j*′_(*t*) for short times compared to γ^–1^, we first expand  around ρ = 1, which leads to eqs 34. Then, we replace each integral of the form ∫dρ *f*(ρ)*G*_*s*_(*t*/τ_*B*_) by (τ_*B*_/*t*)[*g*_*s*_(0) – *g*_*s*_(*t*/τ_*B*_)]*f*(⟨ρ⟩_*s*_).

It is natural to ask about the limit of applicability of this approximation. Unlike fast diffusion, effective diffusion results from the *R* → ∞ limit when Φ_*i*′*j*′_(*t*, ρ) → δ(ρ – β). Therefore, to answer this question, we compare the standard deviations of Φ_*i*′*j*′_(*t*, ρ) and *G*_*s*_^*n*^(*t*,ρ). To simplify this problem, we only consider the leading term of the asymptotic expansion for *t* → ∞. For Φ_*i*′*j*′_(*t*, ρ), it is  (see eq A20 in the Supporting Information). In the case of *G*_*s*_^*n*^(*t*,ρ), all moments  decay with time as , therefore the same applies to the square of the standard deviation σ_*s*_^2^. The transition from the fast diffusion regime *t* < *t*_*t*_ to the effective diffusion regime *t* > *t*_*t*_ occurs at *t*_*t*_ defined by the equation . If we ignore the amplitude of the  term, the transition time is
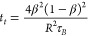
45When τ_*B*_ → 0, the fast diffusion regime extends over the entire time interval because *t*_*t*_ → ∞. Similarly, the effective diffusion regime extends over the entire time interval when *R* → ∞ because *t*_*t*_ → 0. However, the above transition does not occur if *D* ≠ 0 because the standard deviation of *G*_*s*_^*n*^(*t*,ρ) does not tend to zero when *t* → ∞.

### 3.5 Pure Diffusion and Hybrid Model

The relation between the reaction rate constants determines the two regimes: *k*_23_ ≪ *k*_32_ for pure diffusion and *k*_32_ ≪ *k*_23_ for the hybrid model.^21^ To discuss them, we first recast eq 17 as follows
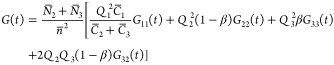
46where the functions *G*_*i*′*j*′_(*t*) are defined by eqs 19. Since (1 – β)*G*_32_(*t*) = β*G*_23_(*t*) and , the cross term is small in both regimes and can therefore be neglected. Thus, in addition to *G*_11_(*t*), either *G*_22_(*t*) or *G*_33_(*t*) contributes to *G*(*t*). When *k*_23_ ≪ *k*_32_, most of the dye molecules are free, and FCS mainly detects these molecules. For *tk*_23_ ≪ 1, *G*_22_(*t*) can therefore be approximated by *G*_*s*_(*t*/τ_*B*_). For long times, , where the effective diffusion time τ_β_ ≈ τ_*B*_ due to small β. This means that *G*_22_(*t*) ≈ *G*_*s*_(*t*/τ_*B*_) for both short and long times, justifying the name “pure diffusion” for this regime.

The hybrid model applies when *k*_32_ ≪ *k*_23_. We studied this model in the Supporting Information for the case of immobile macromolecules. In this limit, *G*_*s*_(*t*/τ_*D*_) = 1, 1/τ_ρ_ = (1 – ρ)/τ_*B*_, and condition (33) is replaced with the inequality
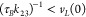
47

From Figure 2, it can be concluded that *v*_*L*_(0) is close to the value *v*_*L*_(0.001), which is 0.48. We calculated *G*_33_(*t*) using the expansion method given by eq 23 and expressed *G*_33_(*t*) as a function of the dimensionless time *t*′ = *k*_32_*t* and the parameters *k*_23_/*k*_32_ and τ_*B*_*k*_23_. We briefly summarize our findings (see the Supporting Information for details). When τ_*B*_*k*_23_ ≳ 1, approximation (23) works very well, but as τ_*B*_*k*_23_ decreases, it deteriorates gradually. However, this deterioration applies to the average *G*_*s*_(*t*/τ_ρ_) values but to a lesser extent to *G*_33_(*t*). As a result, the agreement of the expansion method with accurate numerical calculations is quite good even for small values of τ_*B*_*k*_23_, which do not satisfy condition (47). This is consistent with the reaction-dominant regime (cf. Section 3.3). The coupling between reaction and diffusion can be neglected when τ_*B*_*k*_23_ ≪ 1. Then, *G*_33_(*t*) ≈ exp(−*k*_32_*t*) due to the decay of the complexes. This observation shows that the autocorrelation function based on approximation (23) can be applied to virtually the entire range of τ_*B*_*k*_23_ values in the case of the hybrid model for immobile binding sites.

## 4 Summary

In this section, we have collected the essential information to facilitate the application of our method. Species *A*, *B*, and *C* denote macromolecules, dyes, and fluorescent complexes, respectively. They take part in the reaction *A* + *B* ⇄ *C*. Species 1, 2, and 3 appear as a result of the transformation of the original RD equations. Species 1 diffuses freely. Species 2 and 3 take part in the reaction 2 ⇄ 3; they correspond to the free and bound states of the dye molecule, respectively. Table 1 shows the diffusion coefficients, quantum yields, and equilibrium concentrations for these two types.

**Table 1:** Relationship between Species *A*, *B*, and *C* and 1, 2, and 3

species	diff. coeff.	Q. yield	equil. concentr.
*A*	*D*	0	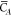
*B*	*D*_*B*_	*Q*_*B*_	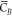
*C*	*D*	*Q*_*C*_	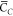
1	*D*	*Q*_1_ = *Q*_*C*_	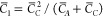
2	*D*_*B*_	*Q*_2_ = *Q*_*B*_	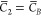
3	*D*	*Q*_3_ = *Q*_*C*_	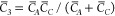

### 4.1 Autocorrelation Function

The autocorrelation function is expressed in terms of components 1, 2, and 3 as follows
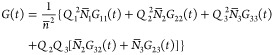
48The symbols used are defined in the tables. For convenience, we recall the exact expressions for *G*_*i*′*j*′_(*t*) (cf. eq 19)
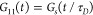
49a
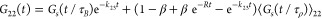
49b
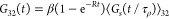
49c
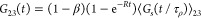
49d
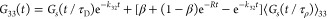
49ewhere .
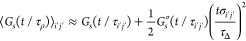
50

The relevant quantities are defined in Tables 2 and 3. The implementation of approximation (50) requires the inverse of the diffusion times τ_22_, τ_33_, and τ_23_ = τ_32_, and standard deviations σ_22_, σ_33_, and σ_23_ = σ_32_. We need the first two moments of each probability density function for this. The autocorrelation function for one-component diffusion, *G*_*s*_(χ), and its second derivative, *G*_*s*_″(χ), are
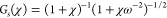
51
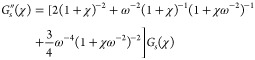
52

**Table 2:** Quantities Related to the Autocorrelation Function^a^

symbol	definition	meaning
*k*_+_, *k*_–_	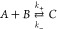	reaction rate constants
*k*_23_	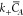	reaction 2 → 3 rate constant
*k*_32_	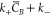	reaction 2 ← 3 rate constant
*R*	*k*_23_ + *k*_32_	chemical relaxation rate
β	*k*_23_/*R*	
γ	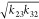	
*H*, *L*		focal volume dimensions
ω	*H*/*L*	aspect ratio
*V*	π^3/2^*L*^2^*H*	effective sampling volume
,	,	avg. no. of molecules in *V*
*n̅*	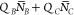	avg. no. of photons in *V*
τ_*D*_	*L*^2^/4*D*	diffusion time of *A* and *C*
τ_*B*_	*L*^2^/4*D*_*B*_	diffusion time of *B*

a We calculate the average values of *G*_*s*_(*t*/τ_ρ_) using approximation (50) (cf. eq. 23).

**Table 3:** Quantities Related to Approximation (50)

symbol	definition	meaning
ρ	0 ≤ ρ ≤ 1	integration variable
*D*_ρ_	(1 – ρ)*D*_*B*_ + ρ*D*	diffusion coefficient
τ_ρ_	*L*^2^/4*D*_ρ_	diffusion time
Φ_*i*′*j*′_(*t*, ρ)	in the Supporting Information	probability density
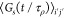	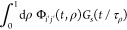	avg. value of *G*_*s*_
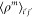	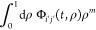	*m*th moment
σ_*i*′*j*′_	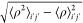	standard deviation
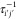	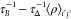	inv. of diffusion time
τ_Δ_^–1^	τ_*B*_^–1^–τ_*D*_^–1^	

Then, we can calculate the average values of *G*_*s*_(*t*/τ_ρ_), which give rise to the functions *G*_22_(*t*), *G*_23_(*t*), *G*_32_(*t*), and *G*_33_(*t*). Finally, the latter functions are inserted into eq 48 to get *G*(*t*). We have described the entire procedure in detail because expressing *G*(*t*) using a single formula would be difficult. However, we can speak of a closed-form expression for the autocorrelation function because it consists only of elementary functions and standard operations.

### 4.2 First Moment, Second Moment, and Standard Deviation

The derivation is provided in the appendix of the Supporting Information.

#### 4.2.1 First Moment


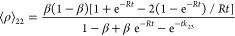
53
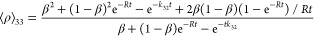
54
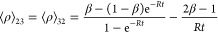
55

#### 4.2.2 Second Moment


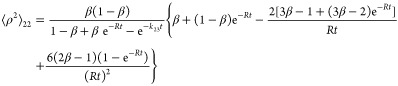
56
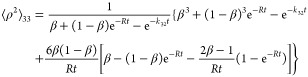
57

#### 4.2.3 Standard Deviation


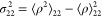
58
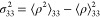
59
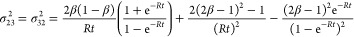
60

The standard deviations σ_22_ and σ_33_ are calculated based on the first and second moments. In the case of σ_23_ = σ_32_, direct computation is easier, as we show in the Supporting Information.

## 5 Discussion and Conclusions

We presented a theoretical study of the fluorescence fluctuation autocorrelation function in the case of a chemical reaction between a macromolecule and a fluorescent dye. The RD equations for the original ternary system are reduced to the case of unimolecular isomerization with different isomer diffusion coefficients. However, instead of isomers, two states of the dye molecule are considered: the free dye and the dye attached to the macromolecule. We found a new solution form of the RD equations useful for deriving an approximation for *G*(*t*) in closed form. The derivation is based on the exact formula for *G*(*t*) that considers the coupling between reaction and diffusion. This coupling takes the form of average values of the autocorrelation function for single-component diffusion, *G*_*s*_(*t*/τ_ρ_). The averaging is over a set of diffusion coefficients parameterized by the dimensionless parameter ρ. Two-component independent diffusion and effective diffusion can be easily reproduced from the general formula. However, calculating the average values of *G*_*s*_(*t*/τ_ρ_) requires an approximation, generally. We used the approximation that assumes that all probability density functions of ρ have one narrow maximum. This assumption is correct when *Rt* is large enough. Therefore, we expanded *G*_*s*_(*t*/τ_ρ_) around the average value of ρ to determine ⟨*G*_*s*_(*t*/τ_ρ_)⟩. However, whether this procedure can be used when the distribution has a broad maximum is not obvious. To answer this question, we found a simple criterion for the applicability of our approximation. Namely, the approximation is valid if the relaxation times of the free and bound dyes are shorter or comparable to the corresponding diffusion times. The numerical studies show that this is the case when *D*/*D*_*B*_ ≲ 1/6. The approximation is also valid for 1/6 < *D*/*D*_*B*_ < 0.3 even when the relaxation times are longer than the diffusion times. We did not study *D*/*D*_*B*_ > 0.3 because assuming the same diffusion coefficients for the macromolecule and the complex is problematic when the difference between *D*_*B*_ and *D* decreases. The RD equations with three different diffusion coefficients should be solved in this case. Interestingly, approximation (23), which was supposed to work well for fast reactions, also turns out to be quite good beyond this range. This behavior may be because the average values and standard deviations of probability density functions are well-defined functions of *Rt* over the entire domain. So, the continuity argument can be used. Moreover, approximation (23) is consistent with the first two terms of the short-time expansion of ⟨*G*_*s*_(*t*/τ_ρ_)⟩. We note that both ⟨ρ⟩ and ⟨ρ^2^⟩ are fractions whose numerator and denominator tend to zero when *Rt* → 0. Although they have finite limits, numerical problems may arise for small values of *Rt*. One should consider this fact when implementing our *G*(*t*) formula.

In our work, we have considered parameter regimes that are relevant experimentally. We use the terminology from ref (21), which was applied to immobile binding sites. The essential elements in our approach are the components of the autocorrelation function *G*(*t*) that depend on two diffusion times and two reaction rate constants. Three dimensionless parameters remain if one of these four parameters is used to define the unit of time. We selected them as τ_*B*_*k*_23_, τ_*D*_*k*_32_, and *k*_23_/*k*_32_. In addition, we introduced the parameter  to divide the parameter space into two regions: *v* < *v*_*L*_ and *v* > *v*_*L*_, where *v*_*L*_ depends on τ_*B*_/τ_*D*_. When *v* < *v*_*L*_, *G*(*t*) obtained by the expansion method should be accurate enough for FCS applications. In this regime, we distinguish between the case of a fast reaction and an intermediate-rate reaction. We make this distinction purely for practical reasons. The approximation of *G*(*t*) in the first case is simpler because it only considers the first moment of the probability density functions. If *v* > *v*_*L*_, it does not automatically mean that the expansion method will fail. This is because our applicability test is based on calculating the average value of *G*_*s*_(*t*/τ_ρ_). If its coefficient is small, the contribution of this average value to *G*(*t*) is negligible. An example is the *k*_32_ ≪ *k*_23_ ≪ τ_*B*_^–1^ regime for immobile binding sites.

Although our model of the autocorrelation function does not cover all interesting cases, it should help determine reaction rate constants and diffusion coefficients using FCS. If the model fits the experimental data, all of the above parameters can be determined. In practice, some of them are known from other experiments, which reduces the number of independent parameters. Usually, the diffusion coefficient of the dye is known. For example, the diffusion coefficient of the dye rhodamine 110, with a hydrodynamic radius of 0.52 nm, is *D*_*B*_ = 470 μm^2^ s^–1^. Then, the diffusion time through a focal volume of size *L* = 0.2 μm is τ_*B*_ = *L*^2^/4*D*_*B*_ = 20 μs.^19^ The diffusion coefficient of a macromolecule is usually several times smaller than *D*_*B*_. If we roughly assume that *k*_23_τ_*B*_ > 1 is consistent with condition (33), then *k*_23_ > 5 × 10^4^ s^–1^ for the binding reaction. We also note that *k*_23_ and *k*_32_ depend on the concentrations of components *A* and *B*. In the case of a fast reaction, the autocorrelation function for effective diffusion is often used to fit FCS data. This choice leads to the effective diffusion coefficient that depends on the equilibrium constant but does not allow the determination of reaction rate constants. In contrast, our approximation for *G*(*t*) should make this possible. In Section 3.3, we assumed that both reaction rate constants were small compared to the corresponding diffusion rates. For short times, first-order approximation in *k*_23_ leads to the autocorrelation function for two-component diffusion complemented by a coupling between reaction and diffusion (see eq 38). This asymptotic form of *G*(*t*) can help determine *k*_23_ if the macromolecule and dye diffusion coefficients are known. The components of *G*(*t*) given by eqs 34 for the range *t* ≪ γ^–1^ show an exponential decay with a time-dependent amplitude. However, according to eqs 19, effective diffusion should prevail when γ^–1^ ≪ *t*. This means that the available time interval in FCS, which, in the case of translational diffusion, is from 10^–5^ s to 1s, should be compared with γ^–1^. The situation is different when macromolecules are immobile. We showed in Section 3.4 that there is a transition from the regime of fast dye diffusion to the regime of effective diffusion, and the transition time is *t*_*t*_ = 4β^2^(1 – β)^2^/(*R*^2^τ_*B*_). This prediction can be verified experimentally if *t*_*t*_ is within the experimental time interval.

Finally, it is worth comparing the problem studied in this article to reaction kinetics modulated by fluctuating environments, e.g., intrachain reactions of polymers in dilute solutions. Since chemical reactions in polymers depend on the polymer conformation, the theory of stochastic processes is used for the second problem.^37^ Conformation-modulated fluorescence emission was investigated, for example, by Yang and Cao^38,39^ (see also references therein). We will focus on two regimes of the lifetime distribution discussed in ref (38). These are the configuration-controlled regime in which reaction kinetics dominate and the diffusion-controlled regime in which conformational relaxation dominates. The diffusive environment is one-dimensional, Markov processes are described by the Smoluchowski equation coupled to a reactive sink, and the diffusion coefficient is *D*. The survival probability in the configuration-controlled regime was calculated using an inhomogeneous first-order cumulant expansion. The survival probability decays nonexponentially in the static limit, defined by *D* → 0. In the diffusion-controlled regime, the reaction is much slower than relaxation, and the system can be well approximated by equilibrium. To this regime, the Wilemski–Fixman approximation was applied.^40,41^ The survival probability decays exponentially in the dynamic limit, defined by *D* → ∞. Despite the apparent differences between the problems mentioned and the methods of solving them, there are some analogies to which we want to draw attention. The effective diffusion limit is analogous to the static limit in ref (38). However, it is not defined by zero diffusion coefficients but by the *R* → ∞ limit. In this limit, the probability density is δ(ρ – β) regardless of time. The configuration-controlled regime corresponds to our fast and intermediate-rate reaction regimes. Then, approximation (23) for the average value of *G*_*s*_(*t*/τ_ρ_) is analogous to the inhomogeneous cumulant expansion in ref (38). To find an analogy with the diffusion-controlled regime, we must assume that the macromolecules are immobile and only consider the dye diffusion coefficient *D*_*B*_. The regime of large *D*_*B*_, discussed in Section 3.4, is called the fast diffusion regime, analogously to the fast reaction regime. When we take the limit *D*_*B*_ → ∞ in eqs 19, this results in the autocorrelation function that decays as exp(−*k*_32_*t*), similar to the dynamic limit in ref (38). In summary, we believe that our research may be of interest not only to researchers using FCS for biophysical problems but also to the broader physical chemistry community.

## Supporting Information Available

Additional mathematical details of our method and its application to the FCS autocorrelation function for immobile macromolecules (PDF)
