# Supplementary material for: Analytical Form of the Fluorescence Correlation Spectroscopy Autocorrelation Function in Chemically Reactive Systems
Source: J Chem Theory Comput. 2024 Mar 22;20(7):2830–41. doi: 10.1021/acs.jctc.3c01176 (PMC11008109; doi:10.1021/acs.jctc.3c01176)
Supplement: Supplementary file 1 — ct3c01176_si_001.pdf [file ct3c01176_si_001.pdf]

Supporting Information to the article:

Analytical form of Fluorescence Correlation Spectroscopy autocorrelation function in chemically reactive systems

Andrzej Poniewierski and Robert Hołyst

Institute of Physical Chemistry, Polish Academy of Sciences Kasprzaka 44/52, 01-224 Warsaw, Poland

* aponiewierski@ichf.edu.pl; * rholyst@ichf.edu.pl

(Dated: March 8, 2024)

I. REACTION-DIFFUSION EQUATIONS

In the system under study, A and B denote macromolecules and fluorescent dyes, respectively. The complex C is a product of the reaction $A + B \rightleftarrows C$, and it is usually also fluorescent. The reaction rate constants are k_+ for the forward reaction and k_- for the backward reaction. In applications to the fluorescence correlation spectroscopy (FCS) [1–6], the reaction-diffusion (RD) equations for the local concentrations, $C_i(\boldsymbol{r}, t)$, are linearised around the equilibrium concentrations, $\bar{C}_i$, where $i = A, B, C$. In open space, the linear equations for concentration fluctuations, $\delta C_i(\boldsymbol{r}, t) = C_i(\boldsymbol{r}, t) - \bar{C}_i$, are customarily transformed to Fourier components, $\delta\tilde{C}_i(\boldsymbol{q}, t)$, where $\boldsymbol{q}$ is the wave vector. $\delta\tilde{C}_i(\boldsymbol{q}, t)$ must satisfy the system of ordinary differential equations

$$\frac{d\delta\tilde{C}_i(\boldsymbol{q}, t)}{dt} = \sum_j M_{ij}(q)\delta\tilde{C}_j(\boldsymbol{q}, t). \tag{1}$$

The matrix below defines the coefficients $M_{ij}(q)$:

$$\boldsymbol{M}(q) = \begin{pmatrix} -D_A q^2 - k_A, & -k_B, & k_C \\ -k_A, & -D_B q^2 - k_B, & k_C \\ k_A, & k_B, & -D_C q^2 - k_C \end{pmatrix}, \tag{2}$$

where $k_A = k_+\bar{C}_B$, $k_B = k_+\bar{C}_A$, $k_C = k_-$ and $q = |\boldsymbol{q}|$. For simplicity, we assume that the diffusion coefficients of the macromolecule and the complex are equal, i.e., $D_A = D_C = D$, where $D < D_B$. We express the general solution of Eq. (1) as $\delta\tilde{C}_i(\boldsymbol{q}, t) = \sum_k \tilde{\mathcal{G}}_{ik}(q, t)\delta\tilde{C}_k(\boldsymbol{q}, 0)$. $\tilde{\mathcal{G}}_{ik}(q, t)$ are Green's functions satisfying the set of equations

$$\frac{d\tilde{\mathcal{G}}_{ik}(q, t)}{dt} = \sum_j M_{ij}(q)\tilde{\mathcal{G}}_{jk}(q, t) \tag{3}$$

with the initial conditions $\tilde{\mathcal{G}}_{ik}(q, 0) = \delta_{ik}$. The standard method for solving Eqs. (1) and (3) is diagonalisation of the matrix $\boldsymbol{M}(q)$ [6]. However, we do not use this method because of a complicated dependence of the eigenvalues and eigenvectors on q. Instead, we transform the concentration fluctuations as follows

$$\delta\tilde{C}_A = \delta\tilde{C}_1 - \delta\tilde{C}_3, \quad \delta\tilde{C}_B = \delta\tilde{C}_2 \quad \delta\tilde{C}_C = (k_A/k_C)\delta\tilde{C}_1 + \delta\tilde{C}_3. \tag{4}$$

The transformation matrix, $T_{ii'}$, is defined by the relations $\delta\tilde{C}_i = \sum_{i'} T_{ii'}\delta\tilde{C}_{i'}$, where $i = A, B, C$ and $i' = 1, 2, 3$. After the transformation, the RD equations assume the following

form

$$\frac{d\delta\tilde{C}_1(\boldsymbol{q},t)}{dt} = -Dq^2\delta\tilde{C}_1(\boldsymbol{q},t), \tag{5a}$$

$$\frac{d\delta\tilde{C}_2(\boldsymbol{q},t)}{dt} = -(D_Bq^2 + k_{23})\delta\tilde{C}_2(\boldsymbol{q},t) + k_{32}\delta\tilde{C}_3(\boldsymbol{q},t), \tag{5b}$$

$$\frac{d\delta\tilde{C}_3(\boldsymbol{q},t)}{dt} = -(Dq^2 + k_{32})\delta\tilde{C}_3(\boldsymbol{q},t) + k_{23}\delta\tilde{C}_2(\boldsymbol{q},t), \tag{5c}$$

where $k_{23} = k_B$ and $k_{32} = k_A + k_C$. The first equation describes free diffusion of component 1. The other two are the RD equations for a binary system whose components participate in a fictitious first-order reaction $2 \rightleftarrows 3$ with reaction rate constants k_{23} and k_{32}. Green's functions transform according to the formula

$$\tilde{\mathcal{G}}_{i'j'}(q,t) = \sum_{ij} T^{-1}_{i'i}\tilde{\mathcal{G}}_{ij}(q,t)T_{jj'}, \tag{6}$$

where $i,j = A,B,C$ and $i',j' = 1,2,3$. $\tilde{\mathcal{G}}_{i'j'}(q,t)$ are Green's functions for Eqs. (5) satisfying the initial conditions $\tilde{\mathcal{G}}_{i'j'}(q,0) = \delta_{i'j'}$. The solution of Eq. (5a) is $\tilde{\mathcal{G}}_{11}(q,t) = \exp(-Dq^2t)$. For convenience, we rewrite Eqs. (5b) and (5c) for Green's functions in the following form

$$\frac{d\tilde{\mathcal{G}}_{2j'}(q,t)}{dt} = -m_2\tilde{\mathcal{G}}_{2j'}(q,t) + k_{32}\tilde{\mathcal{G}}_{3j'}(q,t), \tag{7a}$$

$$\frac{d\tilde{\mathcal{G}}_{3j'}(q,t)}{dt} = -m_3\tilde{\mathcal{G}}_{3j'}(q,t) + k_{23}\tilde{\mathcal{G}}_{2j'}(q,t), \tag{7b}$$

where $m_2 = D_Bq^2 + k_{23}$ and $m_3 = Dq^2 + k_{32}$. When $q = 0$, solving Eqs. (7) is trivial. The solutions are

$$\tilde{\mathcal{G}}_{22}(0,t) = 1 - \beta + \beta e^{-Rt}, \tag{8a}$$

$$\tilde{\mathcal{G}}_{32}(0,t) = \beta(1 - e^{-Rt}), \tag{8b}$$

for $j' = 2$ and

$$\tilde{\mathcal{G}}_{23}(0,t) = (1-\beta)(1 - e^{-Rt}), \tag{9a}$$

$$\tilde{\mathcal{G}}_{33}(0,t) = \beta + (1-\beta)e^{-Rt}, \tag{9b}$$

for $j' = 3$, where $R = k_{23} + k_{32}$ and $\beta = k_{23}/R$. When $q \neq 0$, the solutions of Eqs. (7) assume the integral form:

$$\tilde{\mathcal{G}}_{22}(q,t) = e^{-m_2t} + \gamma\int_0^t d\tau I_1\left[2\gamma\sqrt{(t-\tau)\tau}\right]\sqrt{\frac{t-\tau}{\tau}}e^{-m_2(t-\tau)-m_3\tau}, \tag{10a}$$

$$\tilde{\mathcal{G}}_{32}(q,t) = k_{23}\int_0^t d\tau I_0\left[2\gamma\sqrt{(t-\tau)\tau}\right]e^{-m_3(t-\tau)-m_2\tau}, \tag{10b}$$

for $j' = 2$ and

$$\tilde{\mathcal{G}}_{23}(q,t) = k_{32}\int_0^t d\tau I_0\left[2\gamma\sqrt{(t-\tau)\tau}\right]e^{-m_2(t-\tau)-m_3\tau}, \tag{11a}$$

$$\tilde{\mathcal{G}}_{33}(q,t) = e^{-m_3 t} + \gamma\int_0^t d\tau I_1\left[2\gamma\sqrt{(t-\tau)\tau}\right]\sqrt{\frac{t-\tau}{\tau}}e^{-m_3(t-\tau)-m_2\tau}, \tag{11b}$$

for $j' = 3$. $I_0(\zeta)$ and $I_1(\zeta)$ are the modified Bessel functions [7], and $\gamma = \sqrt{k_{23}k_{32}}$. In general, $I_n(\zeta)$ is a solution of the modified Bessel equation

$$\zeta^2 I_n''(\zeta) + \zeta I_n'(\zeta) - (\zeta^2 + n^2)I_n(\zeta) = 0. \tag{12}$$

It is worth noting that the modified Bessel functions also appear in the context of the FRAP method [8–11]. When n is an integer

$$I_n(\zeta) = \frac{1}{\pi}\int_0^\pi e^{\zeta\cos\theta}\cos(n\theta)d\theta, \tag{13}$$

hence $I_1(\zeta) = I_0'(\zeta)$. Applying the last relation and the modified Bessel equation for $I_0(\zeta)$ shows that Eqs. (10) and (11) do define solutions of Eqs. (7) satisfying the initial conditions $\tilde{\mathcal{G}}_{i'j'}(q,0) = \delta_{i'j'}$. At $q = 0$, these solutions must be compatible with Eqs. (8) and (9), respectively, leading to the following identities

$$\gamma\int_0^t d\tau I_1\left[2\gamma\sqrt{(t-\tau)\tau}\right]\sqrt{\frac{t-\tau}{\tau}}e^{-k_{23}(t-\tau)-k_{32}\tau} = 1-\beta+\beta e^{-Rt} - e^{-k_{23}t}, \tag{14a}$$

$$k_{23}\int_0^t d\tau I_0\left[2\gamma\sqrt{(t-\tau)\tau}\right]e^{-k_{32}(t-\tau)-k_{23}\tau} = \beta(1-e^{-Rt}) \tag{14b}$$

and

$$k_{32}\int_0^t d\tau I_0\left[2\gamma\sqrt{(t-\tau)\tau}\right]e^{-k_{23}(t-\tau)-k_{32}\tau} = (1-\beta)(1-e^{-Rt}), \tag{15a}$$

$$\gamma\int_0^t d\tau I_1\left[2\gamma\sqrt{(t-\tau)\tau}\right]\sqrt{\frac{t-\tau}{\tau}}e^{-k_{32}(t-\tau)-k_{23}\tau} = \beta + (1-\beta)e^{-Rt} - e^{-k_{32}t}. \tag{15b}$$

Probability density functions

The dependence of Green's functions on q is Gaussian, and all integrands in Eqs. (10) and (11) are positive. Therefore we can express $\tilde{\mathcal{G}}_{22}$, $\tilde{\mathcal{G}}_{32}$, $\tilde{\mathcal{G}}_{23}$ and $\tilde{\mathcal{G}}_{33}$ as follows

$$\tilde{\mathcal{G}}_{22}(q,t) = e^{-(D_B q^2 + k_{23})t} + \left(1-\beta+\beta e^{-Rt} - e^{-k_{23}t}\right)\langle e^{-q^2 D_\rho t}\rangle_{22}, \tag{16a}$$

$$\tilde{\mathcal{G}}_{32}(q,t) = \beta\left(1-e^{-Rt}\right)\langle e^{-q^2D_\rho t}\rangle_{32} \tag{16b}$$

and

$$\tilde{\mathcal{G}}_{23}(q,t) = (1-\beta)\left(1-e^{-Rt}\right)\langle e^{-q^2D_\rho t}\rangle_{23}, \tag{17a}$$

$$\tilde{\mathcal{G}}_{33}(q,t) = e^{-(Dq^2+k_{32})t} + \left[\beta + (1-\beta)e^{-Rt} - e^{-k_{32}t}\right]\langle e^{-q^2D_\rho t}\rangle_{33}, \tag{17b}$$

where

$$\langle e^{-q^2D_\rho t}\rangle_{i'j'} = \int_0^1 \Phi_{i'j'}(t,\rho)e^{-q^2D_\rho t}d\rho. \tag{18}$$

$\Phi_{i'j'}(t,\rho)$ are probability density functions of the variable ρ. The diffusion coefficient $D_\rho = D_B(1-\rho) + D\rho$ can take any value between the diffusion coefficients of the macromolecule and the dye. For $q = 0$, we recover Eqs. (8) and (9). When $D_B = D$,

$$\tilde{\mathcal{G}}_{22}(q,t) = \left(1-\beta+\beta e^{-Rt}\right)e^{-q^2Dt}, \tag{19a}$$

$$\tilde{\mathcal{G}}_{32}(q,t) = \beta\left(1-e^{-Rt}\right)e^{-q^2Dt} \tag{19b}$$

and

$$\tilde{\mathcal{G}}_{23}(q,t) = (1-\beta)\left(1-e^{-Rt}\right)e^{-q^2Dt}, \tag{20a}$$

$$\tilde{\mathcal{G}}_{33}(q,t) = \left[\beta + (1-\beta)e^{-Rt}\right]e^{-q^2Dt}. \tag{20b}$$

In this case, Green's functions are the same as for unimolecular isomerisation with equal diffusion coefficients of the isomers.

To determine $\Phi_{i'j'}(t,\rho)$, we first change the integration variable from τ to $t-\tau$ in Eqs. (10b) and (11b). Then $\rho = \tau/t$ is the new integration variable in Eqs. (10) and (11). Hence,

$$\tilde{\mathcal{G}}_{22}(q,t) = e^{-m_2t} + \gamma t\int_0^1 d\rho I_1\left[2\gamma t\sqrt{(1-\rho)\rho}\right]\sqrt{\frac{1-\rho}{\rho}}e^{-t[m_2(1-\rho)+m_3\rho]}, \tag{21a}$$

$$\tilde{\mathcal{G}}_{32}(q,t) = k_{23}t\int_0^1 d\rho I_0\left[2\gamma t\sqrt{(1-\rho)\rho}\right]e^{-t[m_2(1-\rho)+m_3\rho]} \tag{21b}$$

and

$$\tilde{\mathcal{G}}_{23}(q,t) = k_{32}t\int_0^1 d\rho I_0\left[2\gamma t\sqrt{(1-\rho)\rho}\right]e^{-t[m_2(1-\rho)+m_3\rho]}, \tag{22a}$$

$$\tilde{\mathcal{G}}_{33}(q,t) = e^{-m_3t} + \gamma t\int_0^1 d\rho I_1\left[2\gamma t\sqrt{(1-\rho)\rho}\right]\sqrt{\frac{\rho}{1-\rho}}e^{-t[m_2(1-\rho)+m_3\rho]}. \tag{22b}$$

In the same way, we change the integration variable in identities (14) and (15). The comparison of Eqs. (16) and (17) with Eqs. (21) and (22), respectively, yields

$$\Phi_{22}(t,\rho) = \frac{k_{32}tP[1; k_{23}t(1-\rho), k_{32}t\rho]}{1-\beta+\beta e^{-Rt}-e^{-k_{23}t}}, \tag{23a}$$

$$\Phi_{32}(t,\rho) = \Phi_{23}(t,\rho) = \frac{RtP[0; k_{23}t(1-\rho), k_{32}t\rho]}{1-e^{-Rt}}, \tag{23b}$$

$$\Phi_{33}(t,\rho) = \frac{k_{23}tP[1; k_{32}t\rho, k_{23}t(1-\rho)]}{\beta+(1-\beta)e^{-Rt}-e^{-k_{32}t}}, \tag{23c}$$

where

$$P(n;\mu,\nu) = e^{-(\mu+\nu)}\left(\frac{\mu}{\nu}\right)^{n/2} I_{|n|}\left(2\sqrt{\mu\nu}\right) \tag{24}$$

is the Skellam distribution of the variable n [13]. The normalisation $\int_0^1 \Phi_{i'j'}(t,\rho)d\rho = 1$ follows from identities (14) and (15). When $Rt \to \infty$, the asymptotic expansion of $I_n(\zeta)$ for $\zeta \to \infty$ applies [7]:

$$I_n(\zeta) \approx \frac{e^{\zeta}}{\sqrt{2\pi\zeta}}\left[1 - \frac{\mu-1}{8\zeta} + \frac{(\mu-1)\mu-9)}{2!(8\zeta)^2} - \frac{(\mu-1)(\mu-9)(\mu-25)}{3!(8\zeta)^3} + \dots\right], \tag{25}$$

where $\mu = 4n^2$. Inserting $\zeta = 2\gamma t\sqrt{\rho(1-\rho)}$ and taking into account only the leading term in expansion (25) gives

$$I_n\left[2\gamma t\sqrt{(1-\rho)\rho}\right] e^{-t[k_{23}(1-\rho)+k_{32}\rho]} \approx \frac{e^{-t\left[\sqrt{k_{23}(1-\rho)}-\sqrt{k_{32}\rho}\right]^2}}{\sqrt{4\pi\gamma t\sqrt{\rho(1-\rho)}}}. \tag{26}$$

The maximum of the exponential factor at $\rho = \beta$ is 1. Hence $\lim_{Rt\to\infty}\Phi_{i'j'}(t,\beta) = \infty$, while $\lim_{Rt\to\infty}\Phi_{i'j'}(t,\rho \neq \beta) = 0$. This means that $\Phi_{i'j'}(t,\rho) \to \delta(\rho-\beta)$ when $Rt \to \infty$. In contrast, when $Rt \to 0$, the form of $\Phi_{i'j'}(t,\rho)$ results from the expansion: $I_0(\zeta) \approx 1 + \zeta^2/4$, for $\zeta \to 0$, hence $I_1(\zeta) \approx \zeta/2$. Then the probability density functions $\Phi^0_{i'j'}(\rho) = \Phi_{i'j'}(0,\rho)$ are

$$\Phi^0_{22}(\rho) = 2(1-\rho), \quad \Phi^0_{23}(\rho) = \Phi^0_{32}(\rho) = 1, \quad \Phi^0_{33}(\rho) = 2\rho. \tag{27}$$

II. AUTOCORRELATION FUNCTION

The autocorrelation function [6] is defined in the main text. We express it in terms of Green's functions:

$$G(t) = \left(\sum_{i=B,C} Q_i\bar{N}_i\right)^{-2} V\int \frac{d^3q}{(2\pi)^3}\, e^{-[L^2(q_x^2+q_y^2)+H^2q_z^2]/4} \sum_{i,j=B,C} Q_iQ_j\bar{N}_j\tilde{\mathcal{G}}_{ij}(q,t), \tag{28}$$

where Q_B and Q_C are the quantum yields of the fluorescent components. The effective sampling volume V is defined as

$$V = \pi^{3/2} L^2 H = (2\pi)^3 \left[\int d^3q e^{-[L^2(q_x^2+q_y^2)+H^2 q_z^2]/4} \right]^{-1}, \tag{29}$$

and $\bar{N}_i = \bar{C}_i V$. The dimensions H and L define the Gaussian approximation for the light intensity distribution in the focal spot. Their aspect ratio $\omega = H/L$ is usually larger than 1. To calculate the integral in Eq. (28), we insert $\tilde{\mathcal{G}}_{ij} = \sum_{i'j'} T_{ii'} \tilde{\mathcal{G}}_{i'j'} T^{-1}_{j'j}$ (cf. Eq. (6)). Using the transformation matrix and its inverse, i.e.

$$\boldsymbol{T} = \begin{pmatrix} 1 & 0 & -1 \\ 0 & 1 & 0 \\ \frac{k_A}{k_C} & 0 & 1 \end{pmatrix}, \quad \boldsymbol{T}^{-1} = \begin{pmatrix} \frac{k_C}{k_{32}} & 0 & \frac{k_C}{k_{32}} \\ 0 & 1 & 0 \\ -\frac{k_A}{k_{32}} & 0 & \frac{k_C}{k_{32}} \end{pmatrix}, \tag{30}$$

we get

$$\begin{aligned} \sum_{i,j=B,C} Q_i Q_j \bar{C}_j \tilde{\mathcal{G}}_{ij} &= \frac{Q_C^2 \bar{C}_C}{1+K\bar{C}_B} \left(K\bar{C}_B \tilde{\mathcal{G}}_{11} + \tilde{\mathcal{G}}_{33} \right) + Q_B^2 \bar{C}_B \tilde{\mathcal{G}}_{22} \\ &+ Q_B Q_C \left(\bar{C}_B \tilde{\mathcal{G}}_{32} + \frac{\bar{C}_C}{1+K\bar{C}_B} \tilde{\mathcal{G}}_{23} \right). \end{aligned} \tag{31}$$

$K = k_+/k_- = \bar{C}_C/\bar{C}_A\bar{C}_B$ is the reaction $A + B \rightleftarrows C$ equilibrium constant. Whereas the equilibrium constant of the reaction $2 \rightleftarrows 3$ is $k_{23}/k_{32} = \bar{C}_3/\bar{C}_2$, where $\bar{C}_2 = \bar{C}_B$ and $\bar{C}_3 = \bar{C}_A\bar{C}_C/(\bar{C}_A + \bar{C}_C)$. We also define the equilibrium concentration $\bar{C}_1 = \bar{C}_C^2/(\bar{C}_A + \bar{C}_C)$. Then $\bar{C}_1 + \bar{C}_3 = \bar{C}_C$, and we can express Eq. (31) in the following form

$$\sum_{i,j=B,C} Q_i Q_j \bar{C}_j \tilde{\mathcal{G}}_{ij} = Q_1^2 \bar{C}_1 \tilde{\mathcal{G}}_{11} + \sum_{i',j'=2,3} Q_{i'} Q_{j'} \bar{C}_{j'} \tilde{\mathcal{G}}_{i'j'}, \tag{32}$$

where $Q_1 = Q_3 = Q_C$ and $Q_2 = Q_B$ are assumed. According to Eqs. (10b) and (11a) $\tilde{\mathcal{G}}_{32}/k_{23} = \tilde{\mathcal{G}}_{23}/k_{32}$, hence $\bar{C}_2 \tilde{\mathcal{G}}_{32} = \bar{C}_3 \tilde{\mathcal{G}}_{23}$. The last relation reduces the number of independent terms in Eq. (32). Combining Eqs. (28) and (32), we express $G(t)$ only in terms of components 1, 2 and 3, i.e.

$$G(t) = \left(Q_1 \bar{N}_1 + Q_2 \bar{N}_2 + Q_3 \bar{N}_3 \right)^{-2} \left[Q_1^2 \bar{N}_1 G_{11}(t) + \sum_{i',j'=2,3} Q_{i'} Q_{j'} \bar{N}_{j'} G_{i'j'}(t) \right], \tag{33}$$

where $N_{i'} = \bar{C}_{i'} V$ and $Q_1\bar{N}_1 + Q_2\bar{N}_2 + Q_3\bar{N}_3 = Q_B\bar{N}_B + Q_C\bar{N}_C$. The functions $G_{i'j'}(t)$ are related to Green's function defined by Eqs. (16) and (17) as follows

$$G_{i'j'}(t) = V \int \frac{d^3q}{(2\pi)^3} \tilde{\mathcal{G}}_{i'j'}(q,t) e^{-[L^2(q_x^2+q_y^2)+H^2 q_z^2]/4}. \tag{34}$$

Finally, we derive the general expressions for $G_{i'j'}(t)$:

$$G_{11}(t) = G_s(t/\tau_D), \tag{35}$$

$$G_{22}(t) = G_s(t/\tau_B)e^{-k_{23}t} + \left(1-\beta+\beta e^{-Rt} - e^{-k_{23}t}\right)\langle G_s(t/\tau_\rho)\rangle_{22}, \tag{36a}$$

$$G_{32}(t) = \beta\left(1-e^{-Rt}\right)\langle G_s(t/\tau_\rho)\rangle_{32}, \tag{36b}$$

$$G_{23}(t) = (1-\beta)\left(1-e^{-Rt}\right)\langle G_s(t/\tau_\rho)\rangle_{23}, \tag{36c}$$

$$G_{33}(t) = G_s(t/\tau_D)e^{-k_{32}t} + \left[\beta+(1-\beta)e^{-Rt} - e^{-k_{32}t}\right]\langle G_s(t/\tau_\rho)\rangle_{33}. \tag{36d}$$

In Eqs. (36), $\tau_\rho^{-1} = (1-\rho)\tau_B^{-1} + \rho\tau_D^{-1}$, $\tau_D = L^2/4D$, $\tau_B = L^2/4D_B$, and $\langle G_s(t/\tau_\rho)\rangle_{23} = \langle G_s(t/\tau_\rho)\rangle_{32}$. The above expressions form the basis of the approximations discussed in the main text. In the next section, we apply them to the case of immobile macromolecules.

III. IMMOBILE MACROMOLECULES

If the macromolecules are immobile, Eqs. (5b) and (5c) assume a slightly simpler form, i.e.

$$\frac{d\delta\tilde{C}_2(\boldsymbol{q},t)}{dt} = -(D_Bq^2 + k_{23})\delta\tilde{C}_2(\boldsymbol{q},t) + k_{32}\delta\tilde{C}_3(\boldsymbol{q},t), \tag{37a}$$

$$\frac{d\delta\tilde{C}_3(\boldsymbol{q},t)}{dt} = -k_{32}\delta\tilde{C}_3(\boldsymbol{q},t) + k_{23}\delta\tilde{C}_2(\boldsymbol{q},t). \tag{37b}$$

We apply Eqs. (21) and (22) to $D = 0$, and next insert them into definition (34). Hence

$$G_{22}(t) = G_s(t/\tau_B)e^{-k_{23}t} + \gamma t\int_0^1 d\rho I_1\left[2\gamma t\sqrt{(1-\rho)\rho}\right]\sqrt{\frac{1-\rho}{\rho}}e^{-tk_\rho}G_s(t/\tau_\rho), \tag{38a}$$

$$G_{32}(t) = k_{23}t\int_0^1 d\rho I_0\left[2\gamma t\sqrt{(1-\rho)\rho}\right]e^{-tk_\rho}G_s(t/\tau_\rho), \tag{38b}$$

$$G_{23}(t) = k_{32}t\int_0^1 d\rho I_0\left[2\gamma t\sqrt{(1-\rho)\rho}\right]e^{-tk_\rho}G_s(t/\tau_\rho), \tag{38c}$$

$$G_{33}(t) = e^{-k_{32}t} + \gamma t\int_0^1 d\rho I_1\left[2\gamma t\sqrt{(1-\rho)\rho}\right]\sqrt{\frac{\rho}{1-\rho}}e^{-tk_\rho}G_s(t/\tau_\rho), \tag{38d}$$

where $k_\rho = (1-\rho)k_{23} + \rho k_{32}$ and $\tau_\rho^{-1} = (1-\rho)\tau_B^{-1}$. $G_s(t/\tau_\rho)$ is the autocorrelation function for single-component diffusion, defined by the formula

$$G_s(t/\tau_\rho) = \left(1+\frac{t}{\tau_\rho}\right)^{-1}\left(1+\frac{t}{\omega^2\tau_\rho}\right)^{-1/2}, \tag{39}$$

where τ_ρ is the diffusion time. The integrals in Eqs. (38) represent the coupling between reaction and diffusion. To compare $G(t)$ with the autocorrelation function of Ref. [12], we leave only the terms related to components 2 and 3 in Eq. (33) and assume $Q_2 = Q_3$. Then

$$G(t) = \bar{N}^{-1}\left[(1-\beta)G_{22}(t) + 2(1-\beta)G_{32}(t) + \beta G_{33}(t)\right], \tag{40}$$

where $\bar{N} = \bar{N}_2 + \bar{N}_3$ and $\beta = k_{23}/(k_{23}+k_{32}) = \bar{C}_3/(\bar{C}_2+\bar{C}_3)$. The contributions of the cross-correlation functions to $G(t)$ are equal because $\bar{C}_2 G_{32}(t) = \bar{C}_3 G_{23}(t)$. Equations (38) and (40) define the full model for $G(t)$. Following [12], we consider four simplified regimes: *pure diffusion* ($k_{23} \ll k_{32}$), *effective diffusion* ($\tau_B \gg 1/k_{23}$), *hybrid model* ($k_{23} \gg k_{32}$) and *reaction dominant* ($\tau_B \ll 1/k_{23}$). The rate constants k_{23} and k_{32} correspond, respectively, to k^*_{on} and k_{off} in Ref. [12]. If we omit the coupling terms in Eqs. (38) then

$$G(t) \approx \bar{N}^{-1}\left[(1-\beta)G_s(t/\tau_B)e^{-k_{23}t} + \beta e^{-k_{32}t}\right]. \tag{41}$$

A. Pure diffusion and reaction dominant regimes

Approximation (41) is consistent with Ref. [12] in both pure diffusion and reaction dominant regimes, provided that $k_{23}t \ll 1$. In the first case, β is small, hence $G(t) \approx \bar{N}^{-1}G_s(t/\tau_B)$. In the second case, $e^{-k_{23}t} \approx 1$ on the time scale of diffusion, hence

$$G(t) \approx \bar{N}^{-1}\left[(1-\beta)G_s(t/\tau_B) + \beta e^{-k_{32}t}\right]. \tag{42}$$

B. Effective diffusion

We discussed this case in the main text in the context of mobile macromolecules. The effective diffusion occurs when $(k_{23}\tau_B)^{-1} + (k_{32}\tau_D)^{-1} \ll 2$. This inequality is equivalent to the condition $\tau_B \gg 1/(2k_{23})$ for immobile molecules. If $tk_{23} \gg 1$ and $tk_{32} \gg 1$, only the coupling terms in Eqs. (38) contribute to $G(t)$. Then $G_{22}(t) \approx (1-\beta)G_s(t/\tau_\beta)$, $G_{32}(t) \approx \beta G_s(t/\tau_\beta)$ and $G_{33}(t) \approx \beta G_s(t/\tau_\beta)$, where $1/\tau_\beta = (1-\beta)/\tau_B$ (see Eqs. (36)). Finally, $G(t) \approx \bar{N}^{-1}G_s(t/\tau_\beta)$ has the form characteristic of single-component diffusion with the effective diffusion coefficient $D_\beta = (1-\beta)D_B = k_{32}D_B/(k_{23}+k_{32})$.

C. Hybrid model

When $k_{23} \gg k_{32}$, $\beta \approx 1$, and according to Eq. (40) $G(t) \approx \bar{N}^{-1} G_{33}(t)$. The approximate autocorrelation function derived in Ref. [12] results from the assumption: $\delta\tilde{C}_2(\boldsymbol{q}, t) \ll \delta\tilde{C}_3(\boldsymbol{q}, t)$ (see Eqs. (37)), hence

$$\frac{d\delta\tilde{C}_3}{dt} = -\frac{D_B q^2 k_{32}}{D_B q^2 + k_{23}} \delta\tilde{C}_3. \tag{43}$$

We denote the solution of Eq. (43) as

$$\tilde{\mathcal{G}}^h_{33}(q, t) = \exp\left(-\frac{D_B q^2 k_{32} t}{D_B q^2 + k_{23}}\right). \tag{44}$$

It differs from the exact expression given by Eq. (22b) for $D = 0$. $\tilde{\mathcal{G}}^h_{33}$ and $\tilde{\mathcal{G}}_{33}$ satisfy the same initial condition at $t = 0$, but at $q = 0$, $\tilde{\mathcal{G}}_{33}(0, t) = \beta + (1-\beta)e^{-Rt}$, whereas $\tilde{\mathcal{G}}^h_{33}(0, t) = 1$. However, this discrepancy is not very significant when $\beta \approx 1$. Inserting $\tilde{\mathcal{G}}^h_{33}(q, t)$ into Eq. (34) yields the autocorrelation function studied in [12], i.e.,

$$G^h_{33}(t) = V \int \frac{d^3q}{(2\pi)^3} \exp\left(-\frac{D_B q^2 k_{32} t}{D_B q^2 + k_{23}}\right) \exp\left[-\frac{L^2}{4}(q_x^2 + q_y^2) - \frac{H^2}{4} q_z^2\right]. \tag{45}$$

When t is large, only small q values contribute significantly to the integral, hence $G^h_{33}(t) \approx G_s(t k_{32}/\tau_B k_{23})$. This result is consistent with the effective diffusion.

Instead of approximation (45), we use the exact expression for $G_{33}(t)$ given by Eq. (38d). It also has an integral form, however, a one-dimensional integral over ρ replaces the three-dimensional integral over the wave vector. It is convenient to recast Eq. (38d) using Eq. (36d), hence

$$G_{33}(t) = e^{-k_{32}t} + \left[\beta + (1-\beta)e^{-Rt} - e^{-k_{32}t}\right] \langle G_s(t/\tau_\rho)\rangle_{33}. \tag{46}$$

Then we approximate $\langle G_s(t/\tau_\rho)\rangle_{33}$ by expanding $G_s(t/\tau_\rho)$ around $\langle\rho\rangle_{33}$. We showed in the main text that this method can be used in the case of mobile macromolecules if

$$(\tau_B k_{23})^{-1} + (\tau_D k_{32})^{-1} < v_L(\tau_B/\tau_D), \tag{47}$$

where $v_L(\tau_B/\tau_D)$ is a numerically determined function. The lowest τ_B/τ_D value considered was 0.001. Extrapolating inequality (47) to $D = 0$ leads to the condition $(\tau_B k_{23})^{-1} < v_L(0) \approx 0.5$. Thus, the expansion method applies to immobile macromolecules if $\tau_B k_{23} \gtrsim 2$. As $\tau_B k_{23}$ decreases, the ρ distribution widens, and this method becomes less accurate. This

behaviour is demonstrated in Figs. S1 and S2, where $G_{33}(t)$ for the hybrid model is plotted for $k_{23}/k_{32} = 10$ and $k_{23}/k_{32} = 100$, respectively. To show the entire period relevant to the FCS experiment, we use $1/k_{32}$ as the time unit. We observe that the expansion method works very well when $\tau_B k_{23} \gtrsim 1$. Then the relative deviation of the approximate autocorrelation function from the exact one, $\Delta_r G_{33} = (G_{33}^a - G_{33}^e)/G_{33}^e$, is very small. The maximum of $|\Delta_r G_{33}|$ increases when $\tau_B k_{23}$ decreases, but it occurs close to the tail of G_{33}. When $\tau_B k_{23} \ll 1$, G_{33} is virtually indistinguishable from $\exp(-k_{32}t)$. This case corresponds to the reaction dominant regime (cf. Eq. (42)).

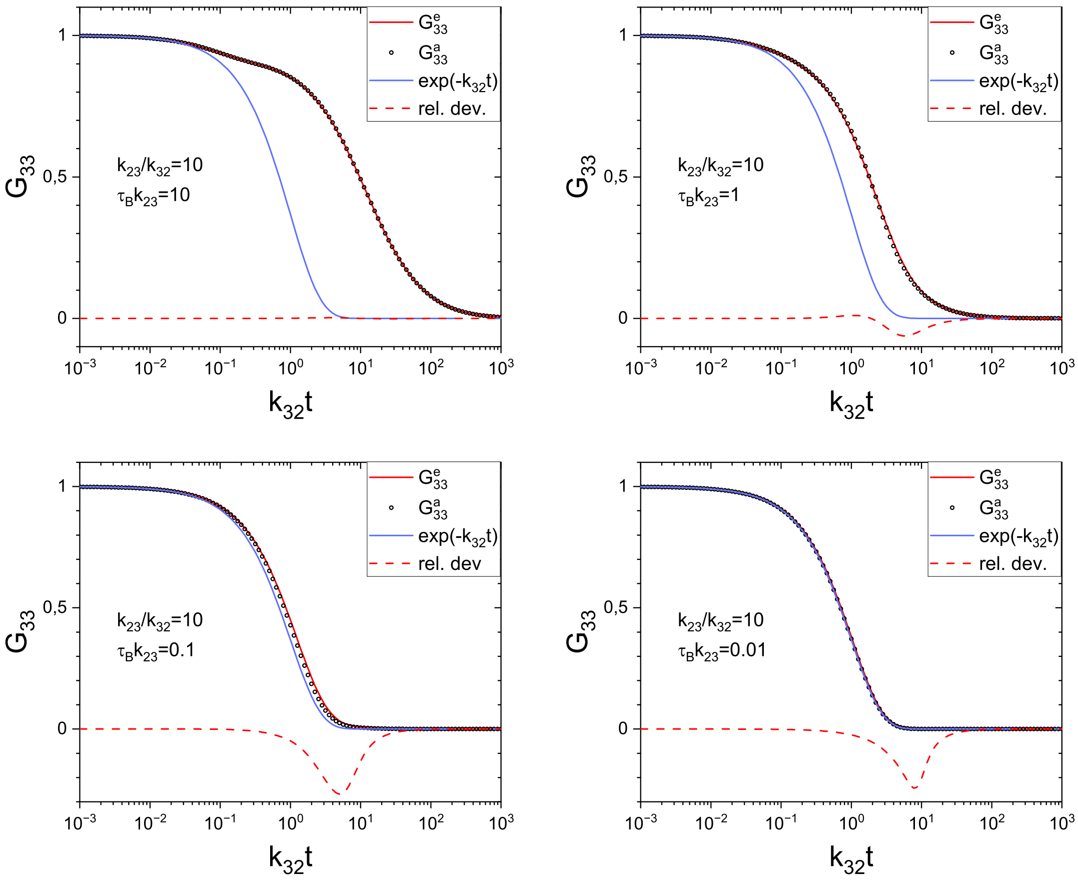

Figure S1. G_{33} for the hybrid model as a function of $k_{32}t$ for $k_{23}/k_{32} = 10$ and a few values of $\tau_B k_{23}$. G_{33}^e (solid red line) is calculated numerically from exact expression (38d). G_{33}^a (circles) is the approximation using the expansion method described in the text. The dashed red line represents the relative deviation, $(G_{33}^a - G_{33}^e)/G_{33}^e$, and the solid blue line shows $\exp(-k_{32}t)$.

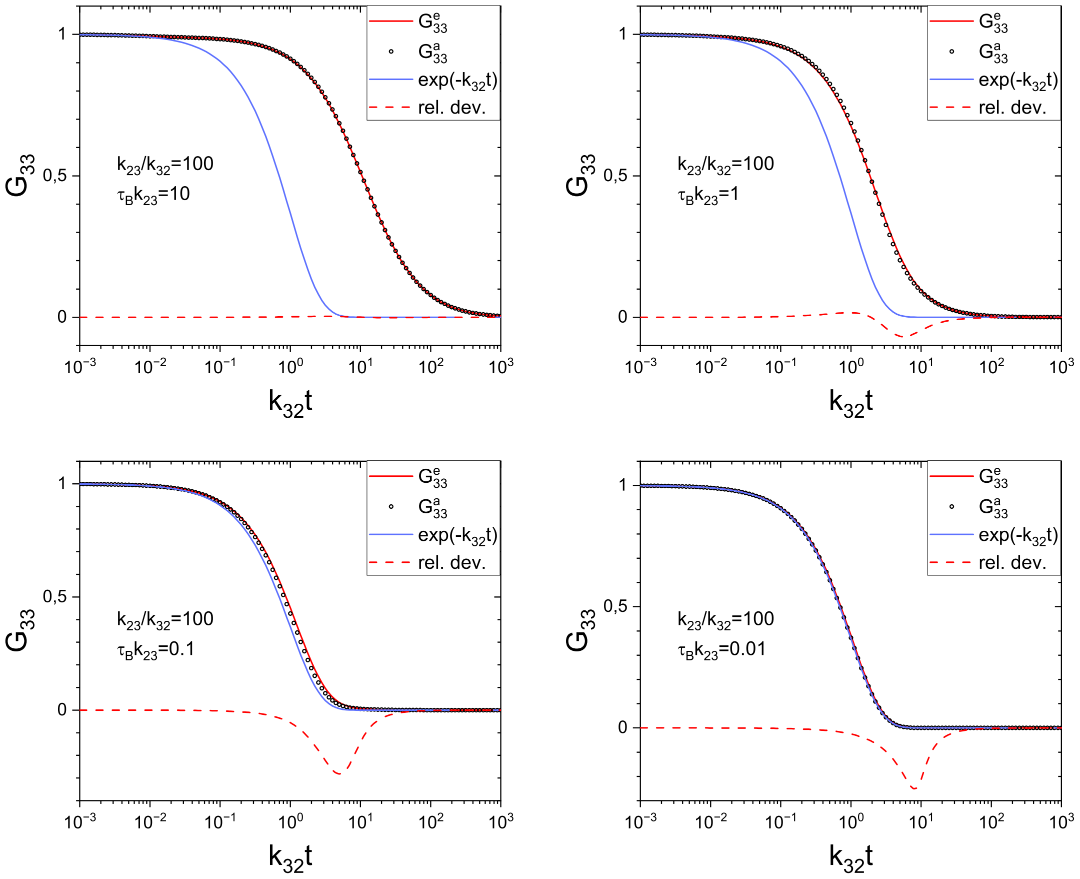

Figure S2. G_{33} for the hybrid model as a function of $k_{32}t$ for $k_{23}/k_{32} = 100$ and a few values of $\tau_B k_{23}$. The meaning of lines and symbols is the same as in Fig. S1.

Appendix A: Moments and cumulants of probability density functions

In this appendix, we derive the general expressions for the moments and cumulants of $\Phi_{i'j'}(t,\rho)$. For simplicity, we will omit the indices. If $\Phi(t,\rho)$ is a probability density function of ρ then the function

$$M(t,s) = \langle e^{s\rho} \rangle = 1 + \sum_{n=1}^{\infty} \mu_n(t) \frac{s^n}{n!} \tag{A1}$$

generates the moments of $\Phi(t,\rho)$,

$$\mu_n(t) = \langle \rho^n \rangle = \left. \frac{\partial^n M(t,s)}{\partial s^n} \right|_{s=0}, \tag{A2}$$

whereas the function

$$\mathcal{K}(t,s) = \ln M(t,s) = \sum_{n=1}^{\infty} \kappa_n(t) \frac{s^n}{n!} \tag{A3}$$

generates the cumulants of $\Phi(t,\rho)$ [14],

$$\kappa_n(t) = \left. \frac{\partial^n \ln M(t,s)}{\partial s^n} \right|_{s=0}. \tag{A4}$$

Cumulants are polynomials of moments, but moments can also be expressed as polynomials of cumulants from the relation

$$\mu_n(t) = \left. \frac{\partial^n \exp[\mathcal{K}(t,s)]}{\partial s^n} \right|_{s=0}. \tag{A5}$$

The relations between the first three cumulants and moments are

$$\kappa_1 = \mu_1, \quad \kappa_2 = \mu_2 - \mu_1^2 = \sigma^2, \quad \kappa_3 = \mu_3 - 3\mu_2\mu_1 + 2\mu_1^3, \tag{A6}$$

$$\mu_1 = \kappa_1, \quad \mu_2 = \kappa_2 + \kappa_1^2, \quad \mu_3 = \kappa_3 + 3\kappa_2\kappa_1 + \kappa_1^3, \tag{A7}$$

where σ is the standard deviation. We can use these general definitions in our problem if we treat $r = k_{23} - k_{32}$ and $\gamma = \sqrt{k_{23}k_{32}}$ as independent parameters. Then we express the reaction rate constants as functions of r and γ, i.e.

$$k_{23} = \frac{1}{2}\left(\sqrt{r^2 + 4\gamma^2} + r\right), \quad k_{32} = \frac{1}{2}\left(\sqrt{r^2 + 4\gamma^2} - r\right), \tag{A8}$$

hence $R = k_{23} + k_{32} = \sqrt{r^2 + 4\gamma^2}$. Multiplying the numerators and denominators by $e^{tk_{23}}$ in definitions (23) gives

$$\Phi_{22}(t,\rho) = A_{22}(t)^{-1} I_1\left[2\gamma t\sqrt{(1-\rho)\rho}\right] \sqrt{\frac{1-\rho}{\rho}} e^{tr\rho}, \tag{A9a}$$

$$\Phi_{23}(t,\rho) = A_{23}(t)^{-1} I_0\left[2\gamma t\sqrt{(1-\rho)\rho}\right] e^{tr\rho} = \Phi_{32}(t,\rho), \tag{A9b}$$

$$\Phi_{33}(t,\rho) = A_{33}(t)^{-1} I_1\left[2\gamma t\sqrt{(1-\rho)\rho}\right] \sqrt{\frac{\rho}{1-\rho}} e^{tr\rho}. \tag{A9c}$$

The normalisation factors in Eqs. (A9) are

$$A_{22}(t) = \int_0^1 I_1\left[2\gamma t\sqrt{(1-\rho)\rho}\right] \sqrt{\frac{1-\rho}{\rho}} e^{tr\rho} d\rho = \frac{(1-\beta)e^{k_{23}t} + \beta e^{-k_{32}t} - 1}{\gamma t}, \tag{A10a}$$

$$A_{23}(t) = \int_0^1 I_0\left[2\gamma t\sqrt{(1-\rho)\rho}\right] e^{tr\rho} d\rho = \frac{e^{k_{23}t} - e^{-k_{32}t}}{Rt}, \tag{A10b}$$

$$A_{33}(t) = \int_0^1 I_1\left[2\gamma t\sqrt{(1-\rho)\rho}\right]\sqrt{\frac{\rho}{1-\rho}}e^{tr\rho}d\rho = \frac{\beta e^{k_{23}t} + (1-\beta)e^{-k_{32}t} - e^{rt}}{\gamma t}. \quad \text{(A10c)}$$

To derive them, we used Eqs. (14) and (15). The probability density functions depend on the parameters γ and r. Using the relations

$$\langle e^{s\rho}\rangle_{i'j'} = \frac{A_{i'j'}(t, r+s)}{A_{i'j'}(t,r)}, \qquad \left.\frac{\partial^n A_{i'j'}(t, r+s)}{\partial s^n}\right|_{s=0} = \frac{\partial^n A_{i'j'}(t,r)}{\partial r^n}, \quad \text{(A11)}$$

we determine the moments and cumulants of $\Phi_{i'j'}(t,\rho)$ as follows

$$\mu_n^{i'j'}(t) = \langle \rho^n \rangle_{i'j'} = A_{i'j'}(t)^{-1}\frac{\partial^n A_{i'j'}(t)}{t^n \partial r^n}, \quad \text{(A12)}$$

$$\kappa_n^{i'j'}(t) = \frac{\partial^n \ln A_{i'j'}(t)}{t^n \partial r^n}. \quad \text{(A13)}$$

The differentiation with respect to r is performed at constant γ. Finally, we derive the expressions for the moments and cumulants using Eqs. (A10), (A12) and (A13):

$$\mu_n^{22} = \frac{t^{-n}(\partial^n/\partial r^n)\left[(1-\beta)e^{tk_{23}} + \beta e^{-tk_{32}} - 1\right]}{(1-\beta)e^{tk_{23}} + \beta e^{-tk_{32}} - 1}, \quad \text{(A14a)}$$

$$\mu_n^{33} = \frac{t^{-n}(\partial^n/\partial r^n)\left[\beta e^{tk_{23}} + (1-\beta)e^{-tk_{32}} - e^{rt}\right]}{\beta e^{tk_{23}} + (1-\beta)e^{-tk_{32}} - e^{rt}}, \quad \text{(A14b)}$$

$$\mu_n^{23} = \mu_n^{32} = \frac{t^{-n}(\partial^n/\partial r^n)\left[(e^{tk_{23}} - e^{-tk_{32}})/R\right]}{(e^{tk_{23}} - e^{-tk_{32}})/R}, \quad \text{(A14c)}$$

and

$$\kappa_n^{22} = t^{-n}\frac{\partial^n}{\partial r^n}\ln\left[(1-\beta)e^{tk_{23}} + \beta e^{-tk_{32}} - 1\right], \quad \text{(A15a)}$$

$$\kappa_n^{33} = t^{-n}\frac{\partial^n}{\partial r^n}\ln\left[\beta e^{tk_{23}} + (1-\beta)e^{-tk_{32}} - e^{rt}\right], \quad \text{(A15b)}$$

$$\kappa_n^{23} = \kappa_n^{32} = t^{-n}\frac{\partial^n}{\partial r^n}\left[\ln(e^{tk_{23}} - e^{-tk_{32}}) - \ln R\right]. \quad \text{(A15c)}$$

The asymptotic decay of cumulants with time follows from the approximation $\kappa_n^{i'j'} \approx t^{-n+1}\partial^n k_{23}/\partial r^n$. In principle, it is possible to calculate any moment and cumulant using Eqs. (A14) and (A15). However, since the computational complexity increases significantly with n, we limit ourselves to the first two moments. The derivatives of k_{23}, k_{32}, R and β with respect to r follow from relations (A8):

$$\frac{\partial k_{23}}{\partial r} = \beta, \quad \frac{\partial k_{32}}{\partial r} = \beta - 1, \quad \text{(A16)}$$

$$\frac{\partial R}{\partial r} = 2\beta - 1, \quad \frac{\partial^2 R}{\partial r^2} = \frac{4\beta(1-\beta)}{R}, \quad \text{(A17)}$$

$$\frac{\partial\beta}{\partial r} = \frac{2\beta(1-\beta)}{R}, \quad \frac{\partial^2\beta}{\partial r^2} = -\frac{6\beta(1-\beta)(2\beta-1)}{R^2}. \tag{A18}$$

Then we use them to derive the first and second moments. We presented the full expressions for the first two moments in the main text. Here we only consider the limits $Rt \to \infty$ and $Rt \to 0$. When $Rt \to \infty$, the leading terms in the asymptotic expansion of $\langle\rho\rangle_{i'j'}$ and $\sigma^2_{i'j'}$ are

$$\langle\rho\rangle_{22} \approx \beta(1-2/Rt), \quad \langle\rho\rangle_{33} \approx \beta+2(1-\beta)/Rt, \quad \langle\rho\rangle_{23} = \langle\rho\rangle_{32} \approx \beta-(2\beta-1)/Rt \tag{A19}$$

and

$$\sigma^2_{i'j'} \approx 2\beta(1-\beta)/Rt \quad \text{for } i', j' = 2, 3. \tag{A20}$$

When $Rt \geq 2$, approximation (A19) is consistent with the conditions $0 \leq \langle\rho\rangle_{i'j'} \leq 1$. The moments of $\Phi_{i'j'}(t,\rho)$ at $t = 0$ are (see Eqs. (27))

$$\langle\rho^n\rangle_{22} = \frac{2}{(n+1)(n+2)}, \quad \langle\rho^n\rangle_{33} = \frac{2}{n+2}, \quad \langle\rho^n\rangle_{23} = \langle\rho^n\rangle_{23} = \frac{1}{n+1}. \tag{A21}$$

Hence

$$\langle\rho\rangle_{22} = 1/3, \quad \langle\rho\rangle_{33} = 2/3, \quad \langle\rho\rangle_{23} = \langle\rho\rangle_{32} = 1/2 \tag{A22}$$

and

$$\sigma^2_{22} = \sigma^2_{33} = 1/18, \quad \sigma^2_{23} = \sigma^2_{32} = 1/12. \tag{A23}$$

---

[1] Magde, D; Elson, E. L; Webb, W. W. Thermodynamic Fluctuations in a Reacting System–Measurement by Fluorescence Correlation Spectroscopy. *Phys. Rev. Lett.* **1972**, *29*, 705–708.

[2] Elson, E. L.; Magde, D. Fluorescence Correlation Spectroscopy. I. Conceptual Basis and Theory. *Biopolimers* **1974**, *13*, 1–27.

[3] Magde, D.; Elson, E. L.; Webb, W. W. Fluorescence correlation spectroscopy. II. An experimental realization. *Biopolimers* **1974**, *13*, 29–61.

[4] Elson, E. L. Fluorescence Correlation Spectroscopy Measures Molecular Transport in Cells. *Traffic* **2001**, *2*, 789–796.

[5] Elson, E. L. Fluorescence Correlation Spectroscopy: Past, Present, Future. *Biophys. J.* **2011**, *101*, 2855–2870.

[6] Krichevsky, O.; Bonnet, G. Fluorescence correlation spectroscopy: the technique and its applications. *Rep. Prog. Phys.* **2002**, *65*, 251–297.

[7] Olver, F. W. J. Bessel Functions of Integer Order. In *Handbook of Mathematical Functions with Formulas, Graphs, and Mathematical Tables*, first edition; Abramowitz, M., Stegun, I. A., Eds.; Applied Mathematics Series 55; United States Department of Commerce, National Bureau of Standards: Washington, D. C., 1964; pp 374–378.

[8] Axelrod, D.; Koppel, D. E.; Schlessinger, J.; Elson, E.; Webb, W. W. Mobility Measurement by Analysis of Fluorescence Photobleaching Recovery Kinetics. *Biophys. J.* **1976**, *16*, 1055–1069.

[9] Soumpasis, D. M. TheoreticaL Analysis of Fluorescence Photobleaching Recovery Experiments. *Biophys. J.* **1983**, *41*, 95–07.

[10] Sprague, B. L.; Pego, R. L.; Stavreva, D. A.; McNally, J. G. Analysis of Binding Reactions by Fluorescence Recovery after Photobleaching. *Biophys. J.* **2004**, *86*, 3473–3495.

[11] Sprague, B. L.; McNally, J. G. FRAP analysis of binding: proper and fitting. *Trends in Cell Biology* **2005**, *15*, 84–91.

[12] Michelman-Ribeiro, A.; Mazza, D.; Rosales, T.; Stasevich, T. J.; Boukari, H.; Rishi, V.; Vinson, C.; Knutson, J. R.; McNally, J. G. Direct Measurement of Assocation and Dissociation Rates of DNA Binding in Live Cells by Fluorescence Correlation Spectroscopy. *Biophys. J.* **2009**, *97*, 337–346.

[13] Skellam, J. G. The frequency distribution of the difference between two Poisson variates belonging to different populations. *J. R. Stat. Soc. Ser. A* **1946**, *109*, 296.

[14] van Kampen, N. G. *Stochastic processes in physics and chemistry*; North-Holland: Amsterdam, 1987.
